# Supplementary material for: The GPS Motif Is a Molecular Switch for Bimodal Activities of Adhesion Class G Protein-Coupled Receptors
Source: Cell Rep. 2012 Aug 30;2(2):321–31. doi: 10.1016/j.celrep.2012.06.015 (PMC3776922; doi:10.1016/j.celrep.2012.06.015)
Supplement: Table S2. Genetic Conservation of GPS: Comparison of Ka/Ks ratios, Related to Figure S4 [file mmc2.pdf]

Table S2. Genetic Conservation of GPS: Comparison of Ka/Ks ratios, Related to Figure S4

**Receptor**	**with HX(S/T)**	**without HX(S/T)**
Celsr1	0.1399	0.12274[#]
Celsr2	0.0337[*]	0.03635[#]
Lphn2	0.0163[$]	0.01641

The evolutionary conservation of the GPS with and without the potential cleavage sequence (HX(S/T)) was compared between orthologs of *Celsr1*, *Celsr2*, and *Lphn2*. The analysis included the coding region of the GPS of *Celsr1*, *Celsr2* and *Lphn2* from 17 vertebrate orthologs (sequences are provided on request).Low Ka/Ks values of the GPS-coding sequences indicate strong purifying (negative or sequence-keeping) selection in all aGPCR investigated. Although there are significant differences of conservation between cleavable (*Celsr2*, *Lphn2*) and non-cleavable (*Celsr1*) GPS (**Celsr2* versus *Celsr1*: p < 10-17; [§]*Lphn2* versus *Celsr1*: p < 10-26), the cleavage sites ([#]with HX(S/T) versus without HX(S/T): p < 10-6) do not contribute to the overall differences in GPS conservations between the aGPCR investigated. Note: no (*Lphn2*) or opposite directions of differences in HXS/T versus without HX(S/T) (*Celsr1*, *Celsr2*) were found.
