# Supplementary material for: The GPS Motif Is a Molecular Switch for Bimodal Activities of Adhesion Class G Protein-Coupled Receptors
Source: Cell Rep. 2012 Aug 30;2(2):321–31. doi: 10.1016/j.celrep.2012.06.015 (PMC3776922; doi:10.1016/j.celrep.2012.06.015)
Supplement: Document S1. Article plus Supplemental Information [file mmc5.pdf]

Cell Reports
Article

Cell PRESS
Open ACCESS

The GPS Motif Is a Molecular Switch for Bimodal Activities of Adhesion Class G Protein-Coupled Receptors

Simone Prömel,[1,3] Marie Frickenhaus,[1,5] Samantha Hughes,[1] Lamia Mestek,[1,6] David Staunton,[1] Alison Woollard,[1] Ioannis Vakonakis,[1] Torsten Schöneberg,[3] Ralf Schnabel,[4] Andreas P. Russ,[1,7] and Tobias Langenhan[1,2,7,*]
[1]Department of Biochemistry, University of Oxford, South Parks Road, OX1 3QU Oxford, UK
[2]Institute of Physiology, Department of Neurophysiology, University of Würzburg, Röntgenring 9, 97070 Würzburg, Germany
[3]Institute of Biochemistry, Molecular Biochemistry, Medical Faculty, University of Leipzig, Johannisallee 30, 04103 Leipzig, Germany
[4]Institut für Genetik, TU Braunschweig, 38106 Braunschweig, Germany
[5]Present address: Max Planck Institute for Molecular Biomedicine, Von-Esmarch-Straße 54, 48149 Münster, Germany
[6]Present address: Institute of Healthy Ageing and Research Department of Genetics, Evolution and Environment, University College London, Gower Street, WC1E 6BT London, UK
[7]These authors contributed equally to this work
*Correspondence: tobias.langenhan@uni-wuerzburg.de
http://dx.doi.org/10.1016/j.celrep.2012.06.015

SUMMARY

Adhesion class G protein-coupled receptors (aGPCR) form the second largest group of seven-transmembrane-spanning (7TM) receptors whose molecular layout and function differ from canonical 7TM receptors. Despite their essential roles in immunity, tumorigenesis, and development, the mechanisms of aGPCR activation and signal transduction have remained obscure to date. Here, we use a transgenic assay to define the protein domains required in vivo for the activity of the prototypical aGPCR LAT-1/Latrophilin in *Caenorhabditis elegans*. We show that the GPCR proteolytic site (GPS) motif, the molecular hallmark feature of the entire aGPCR class, is essential for LAT-1 signaling serving in two different activity modes of the receptor. Surprisingly, neither mode requires cleavage but presence of the GPS, which relays interactions with at least two different partners. Our work thus uncovers the versatile nature of aGPCR activity in molecular detail and places the GPS motif in a central position for diverse protein-protein interactions.

INTRODUCTION

Adhesion G-protein-coupled receptors (aGPCR) form the second largest of the five heptahelical (i.e., spanning seven transmembranes; 7TM) receptor families (Figures 1A and S1A) (Harmar, 2001; Fredriksson et al., 2003a, 2003b). The receptor class is essential for neuronal development, planar cell and tissue polarity as outlined by mutations throughout the spectrum of multicellular organisms. In humans, aGPCR mutations inflict bilateral frontoparietal polymicrogyria (BFPP) and Usher syndrome type IIc (Piao et al., 2004; Weston et al., 2004). In addition, aGPCR have also been implicated in the pathogenesis of attention deficit and hyperactivity disorder (ADHD) (Arcos-Burgos et al., 2010). Although it is accepted that cell-cell or cell-matrix contact mediated by the extracellular adhesion domains results in receptor signaling (Hamann et al., 1996; Stacey et al., 2003), the molecular mechanism of aGPCR activation has remained undetermined (Figure 1A). Prevailing models of aGPCR activation and signaling have emerged from several lines of evidence. First, aGPCR can form distinct multicomponent signaling complexes with other transmembrane molecules in *cis* and in *trans* in an asymmetric layout across a single cell. This mode of signaling has been shown to occur for the cadherin-like aGPCR CELSR/Flamingo (Fmi) of *Drosophila melanogaster* (Usui et al., 1999; Chen et al., 2008). Second, in addition to ligand binding at the N-terminal adhesion domains interaction of a second ligand with the hormone receptor motif (HRM), a domain conserved in many but not all aGPCR, is required for signaling (Shima et al., 2004; Kimura et al., 2006). Third, the "split personality" receptor model proposes that N termini of aGPCR are liberated from their 7TM-module posttranslationally via cleavage at the GPS. The ectodomain fragment is then able to recombine with 7TM-modules of other aGPCR at the membrane (Silva et al., 2009).

In this study we have addressed these working models in the prototypical aGPCR *lat-1*, the nematode homolog of Latrophilin/CIRL/LPHN/CL, in an in vivo setting in *Caenorhabditis elegans*.

RESULTS

lat-1 Is Required for Sperm Function

We have previously selected *lat-1*, the *C. elegans* homolog of Latrophilin/CIRL/LPHN/CL (Figure S1B), for investigations into the physiological requirements of a prototype aGPCR (Figures 1A and S1B). The null allele *lat-1(ok1465)* causes a distinct tissue polarity defect, which is associated with highly penetrant developmental lethality (Langenhan et al., 2009). During the screen for postembryonic phenotypes in *lat-1(ok1465)* mutants we

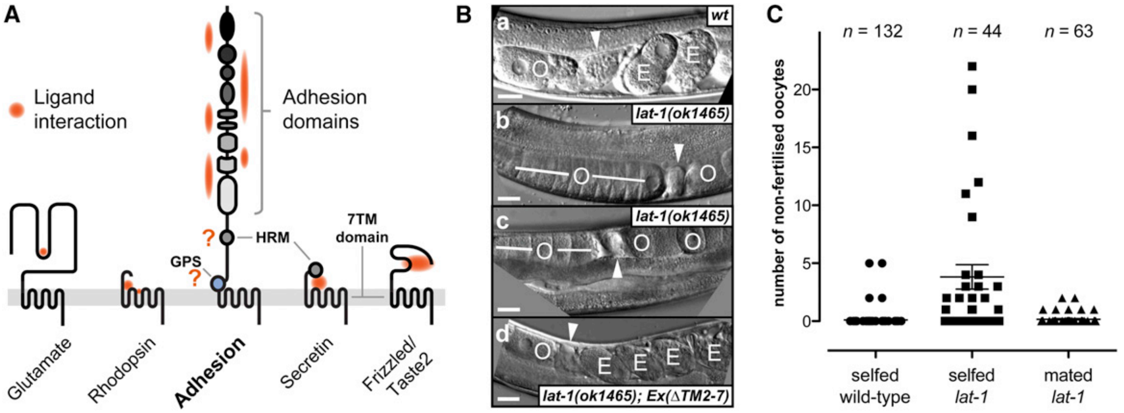

Figure 1. 7TM Families and Fertilization Defects of *lat-1* Mutants

(A) Confirmed and putative (indicated by question marks) sites of ligand interaction for the five 7TM receptor families. See also Figure S1.

(B) Gonads of a wild-type N2 and *lat-1* mutant hermaphrodites; (a) oocytes (O) pass through the spermatheca (arrowhead), become fertilized and embryogenesis ensues. Embryos (E) are visible in the uterus after spermatheca passage. (b,c) *lat-1(ok1465)* mutant hermaphrodite uteri contain a significant fraction of unfertilized oocytes, whereas oocytes in the gonads appear stacked. (d) The fertility defect of *lat-1(ok1465)* hermaphrodites can be rescued by a ΔTM2-7 transgene and by wild-type sperm from mating with wild-type males. Scale bars represent 10 μm.

(C) Quantification of unfertilized oocytes from different genotypes. Experimental error indicated as SEM.

discovered a second component of the Lat phenotype owing to a novel function of *lat-1* in the male germline.

Although more than 70% of *lat-1(ok1465)* embryos and larvae fail to develop due to morphological defects, mutant hermaphrodites surviving to adulthood lay a reduced number of fertilized eggs (N2: 228 ± 3.6, n = 177; *lat-1(ok1465)*: 117 ± 4.0, n = 166; $p < 0.0001$) and an increased number of unfertilized oocytes (N2: 1.6% ± 0.4%, n = 87; *lat-1(ok1465)*: 18.5% ± 1.9%, n = 81; $p < 0.0001$; Figures 1B and 1C). Expression of transgenic constructs are capable of rescuing polarity phenotypes caused by *lat-1(ok1465)*. This rescue can be read out through the level of developmental lethality in the progeny of transformants (Langenhan et al., 2009). Similarly, transgenic transformation with wild-type *lat-1* or *lat-1::gfp* in-frame fusion transgenes rescues fertility demonstrating that *lat-1* is required for reproduction (Figures 1B, 1C, and Table S1).

We employed mating experiments and feminization mutants to identify the gamete type depending on *lat-1* function. In this instance, we observed that the ability to fertilize wild-type oocytes is reduced in *lat-1* mutant males (N2 male x *fem-1* hermaphrodite: 230 ± 8, n = 8; *lat-1(ok1465)* male x *fem-1* hermaphrodite: 193 ± 5, n = 8; p = 0.0012; Figure 1C). This indicates that in addition to defects in embryonic tissue polarity *lat-1* mutants show impaired sperm development or sperm function.

Thus, in addition to embryogenesis sperm function similarly depends on the presence of intact *lat-1* signals offering a second biological context for testing latrophilin activity using modified receptor variants. We have used two *lat-1* phenotypes—development and fertility—to systematically investigate the requirements of protein domains present in the LAT-1 receptor molecule via transgene complementation, and to delineate the contribution of these domains to the *lat-1* wild-type gene product's activity.

The HRM Is Not Required for Receptor Activity

Previously we determined that transgenic rescue of *lat-1(ok1465)* absolutely requires the presence of the lectin-like RBL domain at the N terminus (ΔRBL and ΔTM2-7/ΔRBL; Figures 2A–2C and Table S1) (Vakonakis et al., 2008; Langenhan et al., 2009). The HRM has been suggested to act as a binding site for a putative secondary ligand in aGPCR signaling (Kimura et al., 2006), which would be consistent with its similarity to the ligand-binding domain of secretin-like GPCR (Figures S1B and S1C). Consequently we generated LAT-1 receptor variants with a modified HRM. Interestingly, activity (Figure 2B and Table S1) and expression (Figures 2D, 2E, S2A, S2B, and S3B) of transgenes that lack the *lat-1* HRM (ΔHRM), or in which it is replaced by a divergent heterologous HRM from the nematode CELSR/Fmi homolog *cdh-6* (HRM^{CDH-6}), is indistinguishable from wild-type constructs.

Molecular models of several HRM of B1/secretin-like GPCR that have recently been solved (Grace et al., 2004; Parthier et al., 2007; Runge et al., 2008) are consistent with the rescue data on the dispensable requirement of the HRM for receptor function. The models reveal a highly conserved domain core similar to the short consensus repeat (SCR fold, also known as Sushi or CCP module) (Norman et al., 1991). Peptide-hormone ligands bind to pockets formed by an N-terminal α helix folding back onto this core. The binding is primarily mediated by hydrophobic patches in the SCR fold (Figures 3A and 3B) (Grace et al., 2004; Parthier et al., 2007). The HRM of aGPCR lack the ligand-binding helix whereas the SCR core is conserved (Figures 3A and 3C). The first disulfide bond, which fixes the ligand-binding helix to the core, and the residues forming the ligand-binding pockets on the SCR are absent (Figures 3A and 3C). In contrast to secretin-like HRM, the N and C termini of the SCR fold are predicted to be located at opposing ends of the structure (Figures 3B and 3C), consistent with an extended conformation as described in non-HRM proteins (Norman et al., 1991).

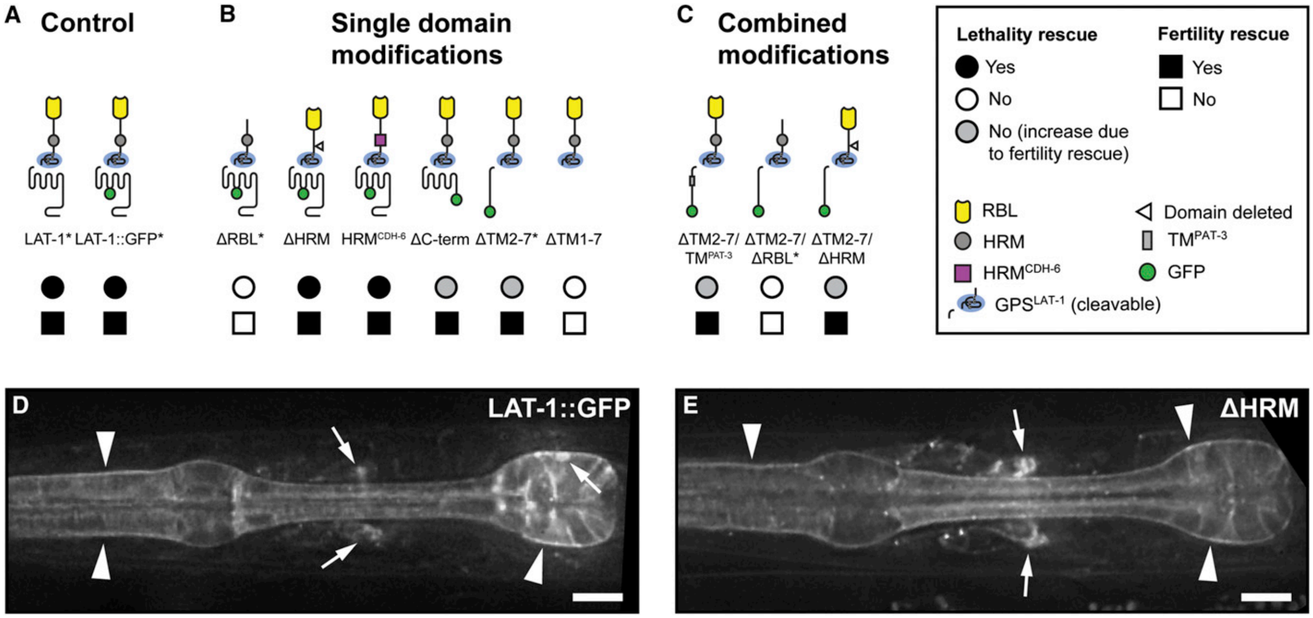

Figure 2. Transgenic Rescue of *lat-1* Mutants Uncover Different Domain Requirements of the LAT-1 Receptor

(A–C) LAT-1 receptor domain modifications exhibit different capacities to rescue lethality (circles) and fertility (boxes) of *lat-1(ok1465)* mutants. Note that the increased number of adult survivors in some transgenic lines is mainly due to increased numbers of eggs laid, whereas these constructs have little effect on the relative rate of developmental failure (gray circles). Previously published constructs are included for comparison and marked by asterisks. For numbers and detailed statistical analysis see Tables S1 and S4.

(D) Deconvolved fluorescence image showing LAT-1::GFP expression at the plasma membrane of pharyngeal muscle cells (arrowheads), neurons in the nerve ring and the pharyngeal nervous system (arrows).

(E) A ΔHRM variant exhibits an expression pattern indistinguishable from the control fusion protein in (D). Scale bars represent 10 μm. See also Figures S2 and S3.

Molecular modeling thus strongly suggests that the HRM of aGPCR are SCR-like structural elements that lack the features required for the binding of peptide hormones by secretin receptor-like HRM (Parthier et al., 2007). We conclude that the HRM is dispensable for productive receptor activation and there is no evidence for an essential ligand-binding activity of the LAT-1 HRM.

LAT-1 Domains Relay Different Functions

We next tested the impact of the 7TM domain of LAT-1, which revealed 7TM-dependent and 7TM-independent components of the Lat phenotype. Transgenes carrying wild-type *lat-1* and *lat-1::gfp* fusions rescue lethality and fertility phenotypes in *lat-1(ok1465)* animals (Figure 2A and Table S1). In contrast, constructs expressing truncated LAT-1 variants lacking the 7TM/C terminus module (ΔTM2-7) or the intracellular C-terminal domain (ΔC-term) retain activity to complement the fertilization defect in *lat-1* mutants, but did not rescue the tissue polarity phenotype (Figure 2B and Table S1).

To rule out that 7TM-independent activity of the ΔTM2-7 receptor requires specific sequences in the first TM helix, we exchanged the remaining TM1 segment of the ΔTM2-7 receptor for a heterologous transmembrane anchor from the nematode integrin homolog *pat-3*. ΔTM2-7/TM^{PAT-3} exhibited no loss of activity compared to a ΔTM2-7 transgene (Figure 2C and Table S1). In contrast, functionality of the ΔTM2-7 transgene was still absolutely dependent on the presence of the RBL domain (ΔTM2-7/ΔRBL; Figure 2C and Table S1) (Langenhan et al., 2009).

Three possible models could explain the 7TM-independent function of LAT-1. First, the ectodomain might act as a ligand for a reciprocal receptor on an adjacent cell to provide a "reverse signaling" function, as has been shown for Notch-Delta signaling (Bray, 2006). Second, the split personality receptor model would suggest that the truncated constructs express an intact ectodomain, which is released by GPS cleavage and recombines with different 7TM domains to form an active receptor (Silva et al., 2009). As a third alternative, the truncated constructs might identify a distinct "forward signaling" state of LAT-1 receptor complex that does not require 7TM domain activation, e.g., the interaction and association with a coreceptor on the LAT-1-expressing cell (Chen et al., 2008; Strutt and Strutt, 2008).

Proteolysis of the GPS Is Dispensable for LAT-1 Activity

We investigated the 7TM-independent function of LAT-1 by mutagenesis of the GPS. The GPS is the most highly conserved feature of aGPCR (Figures 1A and S1B), and is invariably present proximal to the 7TM domain (Figure S1C). The polycystic kidney disease gene product Polycystin-1 (PKD1) belongs to the only other protein family containing a GPS; in this protein context the GPS is also positioned close to a transmembrane domain (Figure S1B). The autocatalytic cleavage activity of the GPS in different molecular contexts has been investigated in detail (Lin et al., 2004; Wei et al., 2007), and crystal structures revealed

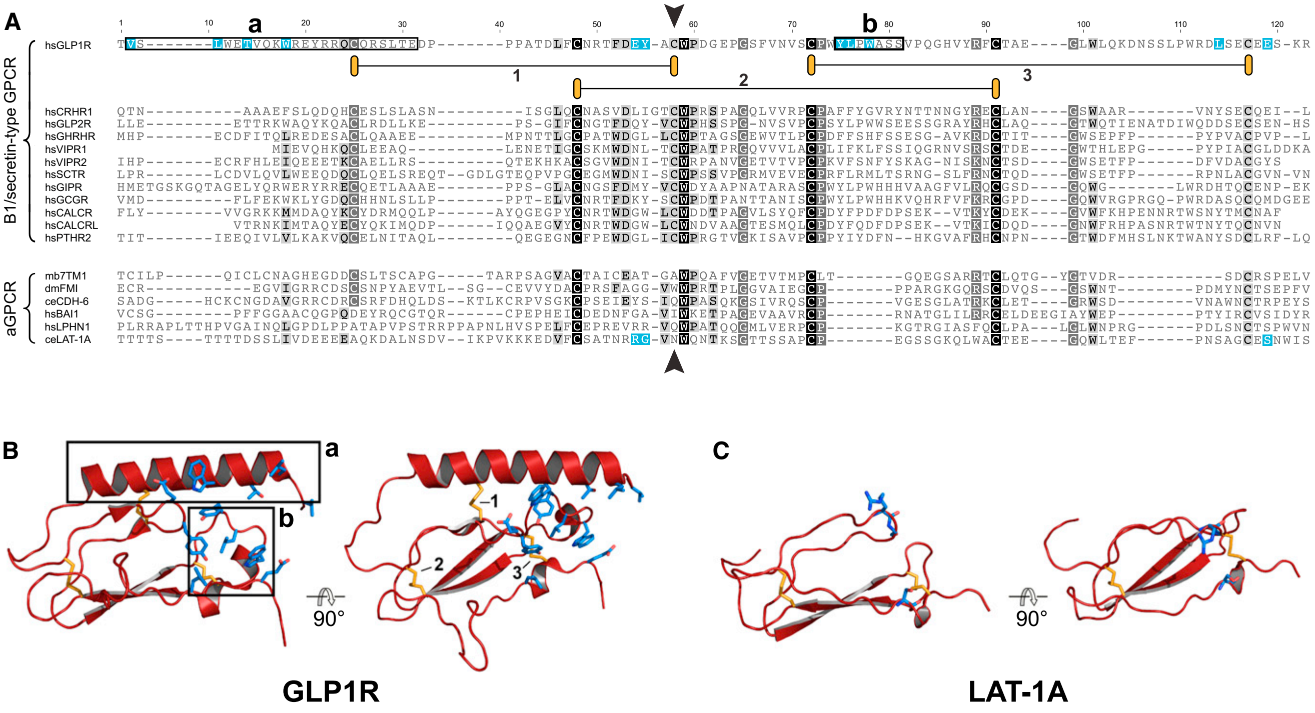

Figure 3. The HRM of the LAT-1 Receptor Is Not Required for Ligand Binding

(A) B1/secretin-type HRM aligned to representative HRM of aGPCR. The Cys residue in position 58 of the alignment is absent in aGPCR HRM (arrowhead). Residues forming the N-terminal α helix (a) and ligand binding pocket (b) in GLP1R are boxed. Disulfide bridges are numbered 1–3 (orange), residues that form the ligand binding interface are labeled in cyan and correspond to structures in (B) and (C). Species abbreviations: ce, *Caenorhabditis elegans*; dm, *Drosophila melanogaster*; hs, *Homo sapiens*; mb, *Monosiga brevicollis*.

(B and C) Orthogonal views of 3D structures of the HRM of human GLP1R (B) (Runge et al., 2008) and a structural model of the *C. elegans* LAT-1 HRM (C) derived from GLP1R data. The N-terminal α helix (a) and the extended loop (b) are boxed in the GLP1R structure (B) whereas they are both absent in the LAT-1A HRM (C).

that the GPS motif is embedded within a larger GAIN (GPCR-autoproteolysis inducing) domain required and sufficient for GPS proteolysis (Araç et al., 2012).

C. elegans LAT-1 and its paralog LAT-2 possess the HL↓(S/T) cleavage consensus sequence and are cleaved as predicted (Figures 4A and S4A). In a split personality receptor model, cleavage of LAT-1 molecules at the GPS is essential for activity by release of the ectodomain and re-association with other 7TM modules.

To probe whether GPS cleavage is required for LAT-1 signaling, we introduced mutations at positions −2 (GPS^{H528A}) or +1 (GPS^{T530A}) of the consensus motif, which abolish autocatalytic cleavage activity but retain cell surface expression (Lin et al., 2004; Yu et al., 2007). As expected, LAT-1 proteins containing either mutation were resistant to autocatalytic cleavage (Figure 4B) but were expressed normally (Figures 4C, S2C, and S3B). Surprisingly, the rescue of lethality and fertility phenotypes by cleavage-deficient full-length receptors is statistically not different from cleavable wild-type controls (Figure 4E and Table S1).

These results are consistent with the observation that the consensus sequence for autocatalytic cleavage of aGPCR is frequently lost in evolution, as has occurred in the CELSR/Fmi, EMR, and BAI aGPCR subfamilies that constitute ~50% of the aGPCR family (Figures S1A and S4B). Analysis of Ka/Ks ratios indicates persistent strong selective pressure on the GPS-coding regions even if the cleavage consensus is lost (Table S2), strongly suggesting a cleavage-independent role of the GPS.

We next introduced noncleavable GPS modifications into the truncated ΔTM2-7 receptor context, showing that the 7TM-independent activity of LAT-1 does also not require GPS cleavage (GPS^{H528A}/ΔTM2-7, GPS^{T530A}/ΔTM2-7; Figure 4F and Table S1). This is further supported by the fact that the truncated constructs display similar activities in *lat-1* single mutants and in *lat-1 lat-2* double mutants (data not shown). Together, these results indicate that the 7TM-independent function cannot be explained by a transfer of the LAT-1 N-terminal fragment (NTF) to the C-terminal fragment (CTF) of LAT-2 (Volynski et al., 2004; Silva et al., 2009).

An Interaction between the GPS and 7TM Domains Is Required for LAT-1 Function

Although autocatalytic cleavage is not absolutely required and conserved, the presence of the GPS immediately next to TM domains is highly specific for aGPCR and PKD1 (Figures 1A and S1B). A secreted version of the LAT-1 ectodomain (ΔTM1-7; Figure 2B) and in-frame deletions of the GPS are completely devoid of rescuing activity in both truncated (ΔGPS/TMΔ2-7) (Langenhan et al., 2009) and full-length context (ΔGPS; Figures

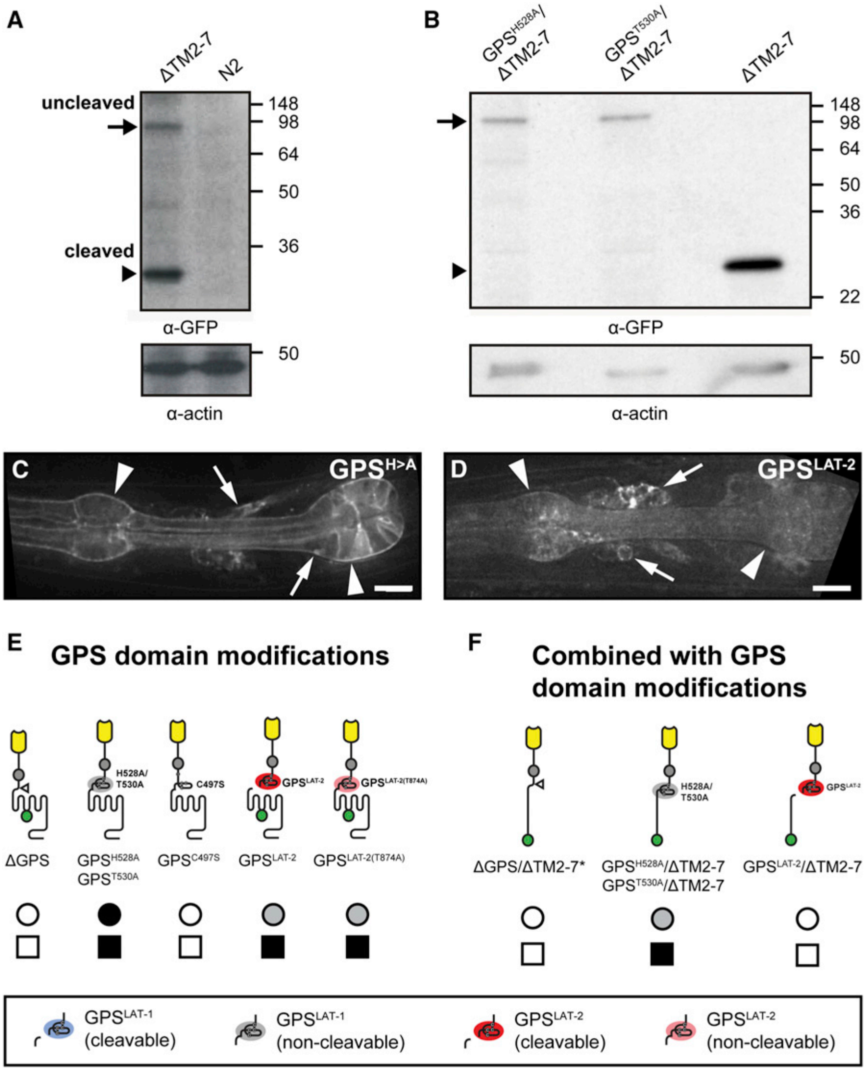

Figure 4. Structure but Not Autocatalytic Function of the GPS Is Required for LAT-1 Activity and Expression

(A) Western blot of a ΔTM2-7::GFP fusion protein extracted from transgenic worm culture (N2: wild-type *C. elegans* control) show that the receptor is cleaved.

(B) Point mutations H528A and T530A abolish cleavage activity. Full-length (arrows) and cleaved proteins (arrowheads). See also Figure S4.

(C and D) Transgenically expressed modified LAT-1::GFP transgene products are delivered to the membrane in neurons (arrows) and pharyngeal muscle cells (arrowheads) independent of GPS cleavage (C) or exchange of the LAT-1 GPS for the LAT-2 GPS. See also Figures S2 and S3. For domain nomenclature see also legend in Figure 2.

(E) Deletion of the GPS and a missense mutation (C497S), which abolishes its structural integrity, abrogate rescuing activity of respective full-length transgenes. In contrast, mutations that leave the GPS structure unaffected possess wild-type rescue function. A heterologous GPS from LAT-2 within the LAT-1 receptor context rescues fertility but not developmental phenotypes of *lat-1 (ok1465)* mutants.

(F) The remaining rescuing function of the LAT-1/LAT-2 chimeric receptor relies on the presence of the 7TM/C terminus module but not its autocatalytic activity. For domain nomenclature see also legend in Figure 2 and Tables S1 and S4.

4E, 4F, and Table S1), whereas surface expression of ΔGPS is intact (data not shown). These results suggest that the GPS is essential for LAT-1 signaling. These findings argue against a reverse signaling model, as the presentation of the RBL domain on a shortened membrane-tethered ectodomain should be sufficient to supply rescue activity and the membrane-proximal GPS should not be required. A clinically relevant mutation inside the GPS of the GPR56 receptor (GPS^{C497S}) is present in patients suffering from bilateral frontoparietal polymicrogyria (BFPP). This mutation changes an absolutely conserved cysteine residue, which is required for receptor maturation and cell surface expression (Piao et al., 2004). We confirmed this in our transgenic assay by the introduction of an equivalent mutation in *lat-1* disrupting the predicted disulfide bond pattern of the GPS, which abrogated LAT-1 surface expression and resulted in an inactive receptor (Figure 4E). Thus, the inactivity of the ΔGPS constructs is due to a sequence-specific requirement for the LAT-1 GPS.

To test for a sequence-specific requirement of the LAT-1 GPS, we constructed chimeric receptors in which the LAT-1 GPS was exchanged for the GPS of *C. elegans* LAT-2 (GPS^{LAT-2}; GPS^{LAT-2}/ΔTM2-7). The primary sequence of the LAT-2 GPS is similar in length and is 50% identical in amino acid sequence to the LAT-1 GPS. It retains the positions and intramolecular distances of all key structural elements (Figure S4A). The chimeric constructs undergo autocatalytic cleavage (data not shown) and show normal expression on the cell surface (Figures 4D, S2D, and S3B). Surprisingly, the full-length GPS^{LAT-2} receptor chimera rescues the fertilization defect of *lat-1(ok1465)* mutants but has no activity in tissue polarity signaling (Figure 4E and Table S1), a profile indistinguishable from constructs lacking an active 7TM domain (Figure 2B and Table S1). In contrast, the truncated chimeric construct GPS^{LAT-2}/ΔTM2-7 shows no residual activity (Figure 4E and Table S1).

This indicates that a sequence-specific function of the GPS other than autocatalytic cleavage or surface expression is required for the 7TM-dependent and 7TM-independent activities of LAT-1.

Cross-Activation of LAT-1 Receptors

All findings regarding the different LAT-1 receptor variants are consistent with a model in which the 7TM-independent function

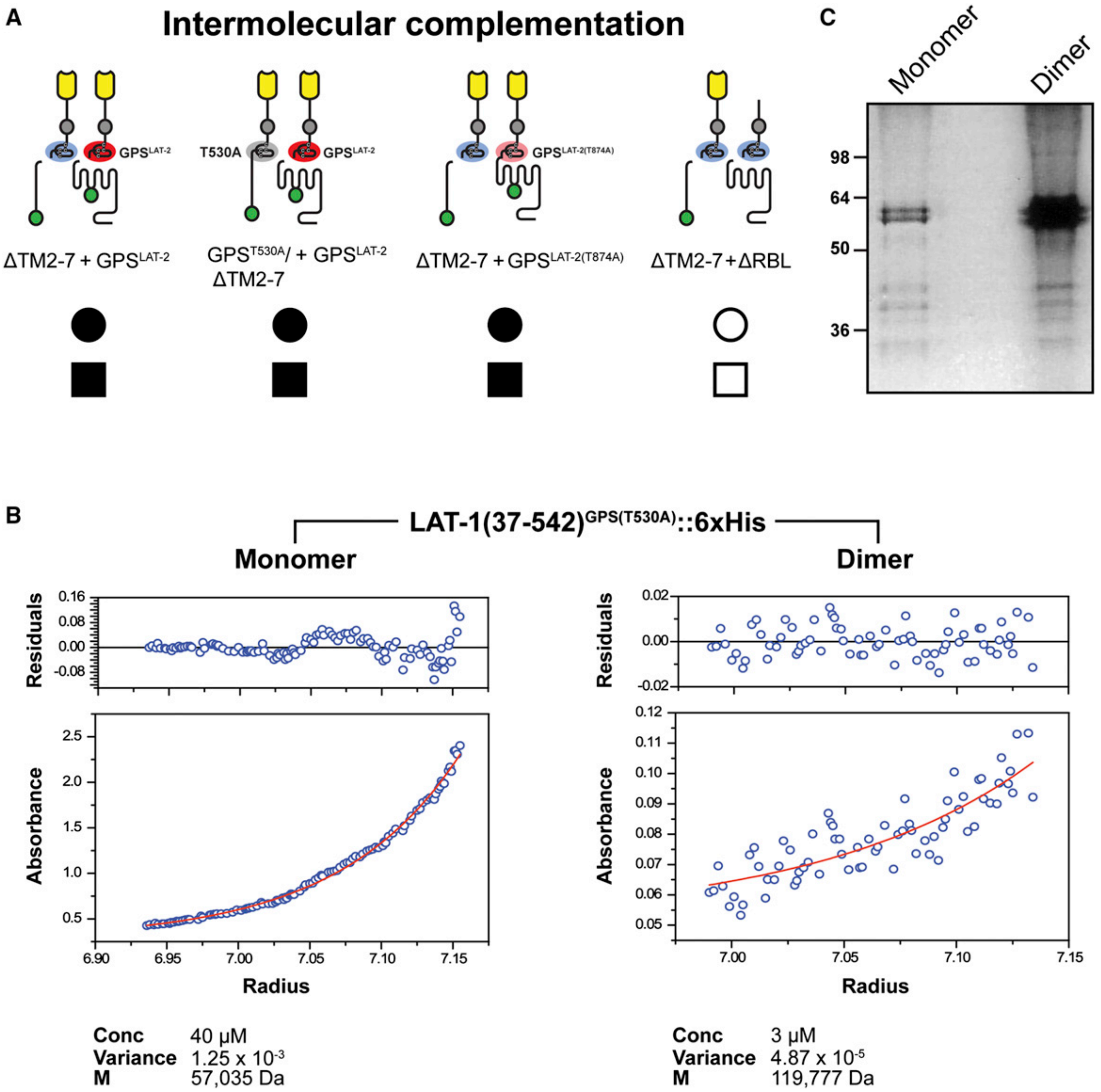

Figure 5. Signaling of the LAT-1 Receptor via Cross-Activation in Dimeric Complex

(A) Intermolecular complementation of the *lat-1(ok1465)* phenotype is fully achieved by pairs of donor (left) and recipient (right) LAT-1 receptors independently of GPS cleavage. The RBL domain is required in both partners. See also Tables S1 and S4.

(B) Analytical ultracentrifugation of LAT-1 ectodomain fractions. The N terminus contained a T530A GPS point mutation disabling cleavage but not impairing function. The receptor can adopt monomeric (left panel) and a tight dimeric form (right panel). The predicted masses of 60 kDa for a monomer and 120 kDa for a dimer were obtained from protein samples at 20°C using sedimentation equilibrium measurements.

(C) A nonreducing polyacrylamide gel reveals a single band (60 kDa) in both monomer and dimer samples indicating that the dimeric form of the LAT-1 N terminus is not covalently linked. Additional bands smaller in size than 60 kDa indicate degraded protein.

is mediated by the localization of LAT-1 in a presignaling complex, which can be mediated by either the 7TM or the GPS. The full receptor function requires the presence of a matching pair of GPS and 7TM domains, for example by the GPS acting as a tethered endogenous ligand for the 7TM domain.

To test this model, we investigated whether a pair of mutant transgenes expressing different receptor variants each lacking the 7TM function can reconstitute full receptor activity by intermolecular complementation (Rivero-Müller et al., 2010). In this assay, the truncated ΔTM2-7 construct provides the wild-type N terminus and GPS, whereas the $GPS^{LAT\text{-}2}$ construct harboring a mutant N terminus contributes a wild-type but inactive 7TM/C terminus module. Coinjection of the transgenes ΔTM2-7 and $GPS^{LAT\text{-}2}$, which individually only rescue the fertility defect but not lethality to wild-type level, fully reconstitutes LAT-1 activity for both requirements (Figure 5A and Table S1). We excluded that DNA recombination between the transgenes accounted for the intermolecular complementation as we were unable to detect repaired constructs by PCR in rescued strains.

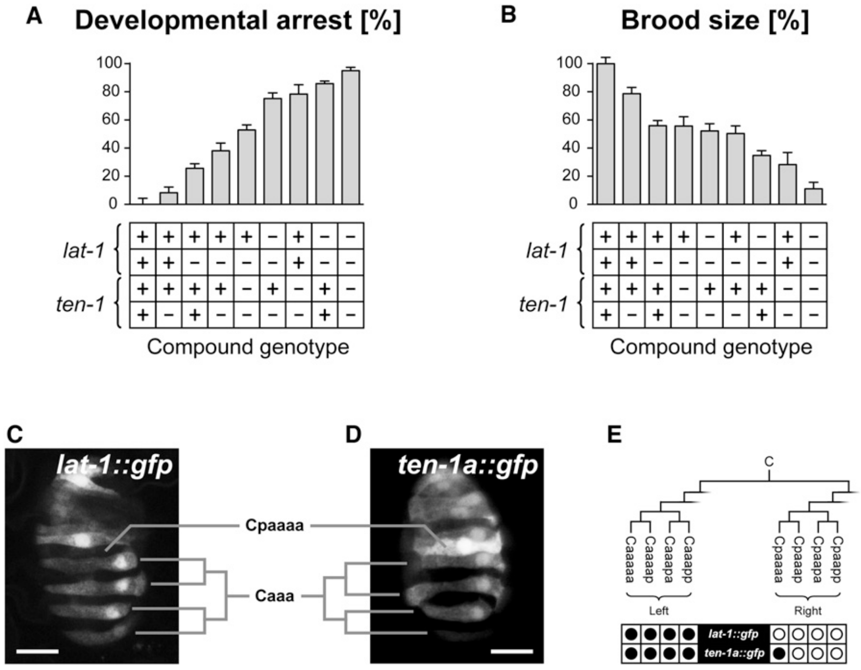

Figure 6. Genetic Interaction and Coexpression of *lat-1* and *ten-1*

(A and B) Frequency of developmental (A) and fertility (B) defects in animals with different dosages of *lat-1* and *ten-1*. Genotypes ordered in ascending severity of phenotype. Experimental error indicated as SEM. See also Table S3.

(C and D) Expression of *lat-1::gfp* (C) and *ten-1a::gfp* (D) transgenes in epidermoblasts derived from the Caaa lineage during dorsal intercalation. Note that in Cpaaaa only *ten-1a::gfp* expression was found. Scale bars represent 10 μm.

(E) Summary of *lat-1::gfp* and *ten-1a::gfp* activity in Caaax and Cpaaax lineages. Expressing cell, filled bullet; nonexpressing cell, open bullet.

We also observed that the intermolecular complementation of LAT-1 function is not dependent on GPS cleavage, as a GPS cleavage mutation on the "donor" (ΔTM2-7/GPS^{T530A}) or the "recipient" (GPS$^{LAT-2(T874A)}$) side does not prevent productive complementation (Figure 5A and Table S1). The fact that the recipient construct can be cleavage-deficient strongly argues against the reconstitution of active monomers by transfer of N termini to 7TM domains in a split personality receptor scenario, as in our assay the inactive N terminus at the recipient receptor was inaccessible for replacement through lack of GPS cleavage.

However, both donor and recipient require the presence of the RBL domain, as the ΔTM2-7 construct does not complement the ΔRBL mutant that contains a wild-type GPS and 7TM (Figure 5A). This suggests that ligand-induced proximity of two LAT-1 receptors mediated by the RBL domain might be required for the intermolecular complementation of GPS and 7TM domains. Our results are consistent with a model in which the ligand-induced dimerization of the NTF leads to the cross-activation of the 7TM domain by the GPS of the partner molecule. To further test this dimerization/activation model, we performed biochemical studies of the LAT-1 ectodomain expressed in HEK293 cells. Gel filtration and analytical ultracentrifugation of supernatants from cells expressing an epitope-tagged LAT-1 ectodomain show that the LAT-1 ectodomain is present in both monomeric and dimeric form (Figure 5B). The dimeric form is very stable in low protein concentrations, but is not mediated by covalent bonds (Figure 5C). These data are consistent with the prediction that the LAT-1 ectodomains can form stable dimers.

lat-1 and *ten-1*/Teneurin Are Expressed on the Same Cells and Are Not Epistatic

Although the ability for intermolecular complementation sheds light on the 7TM-dependent activity of LAT-1, it does not account for the 7TM-independent function of the receptor. Such function might require interaction with an additional molecule(s) to yield co-reception and/or co-signaling similar to the association of the aGPCR *Fmi*/*Stan* of *Drosophila melanogaster* with the transmembrane proteins *Frizzled* (*Fz*) and *Van Gogh*/*Strabismus* (*Vang*/*Stbm*) (Chen et al., 2008; Strutt and Strutt, 2008).

In a recent biochemical screen Teneurin-2/Lasso/Neurestin/DOC4/Tenascin-m was identified as a potential ligand for the Latrophilin-1 (Silva et al., 2011). The *C. elegans* genome contains only a single teneurin homolog, *ten-1*. Animals carrying the null allele *ten-1(ok641)* exhibit morphogenesis defects (Drabikowski et al., 2005) phenocopying defects observed in *lat-1(ok1465)* embryos. Consequently, we sought to confirm whether *lat-1* and *ten-1* function as receptor-ligand pair in vivo by investigating the genetic relationship between the null alleles *lat-1(ok1465)* and *ten-1(ok641)* on the basis of their individual and combined effects on development and fertility (Langenhan et al., 2009).

Interestingly, *lat-1; ten-1* double mutants exhibited almost completely penetrant developmental arrest and a greatly reduced brood size (Figures 6A, 6B and Table S3). We further observed that *lat-1* and *ten-1* alleles displayed nonallelic noncomplementation as animals carrying at least one wild-type allele for both loci already exhibited significant defects in embryogenesis and fertility (Figures 6A, 6B and Table S4). This dosage-sensitivity indicates that both genes are acting at least partly in parallel during development and germline function and implies a synergistic rather than linear interaction between both genes. Although the devastating condition of the *lat-1; ten-1* double mutants precluded the introduction of the ΔTM2-7 receptor as a direct test of whether the 7TM-independent activity of LAT-1 requires presence of TEN-1, our genetic data are consistent with a model where both proteins function as coreceptors for a yet unknown ligand relaying nonidentical signaling outputs into the same cell.

To corroborate this model we studied the expression patterns of *lat-1* and *ten-1* using transcriptional reporter transgenes. We have previously noted that an integrated *lat-1::gfp* transcriptional reporter transgene is expressed during epidermal dorsal intercalation of the epidermis, specifically only in left epidermoblasts

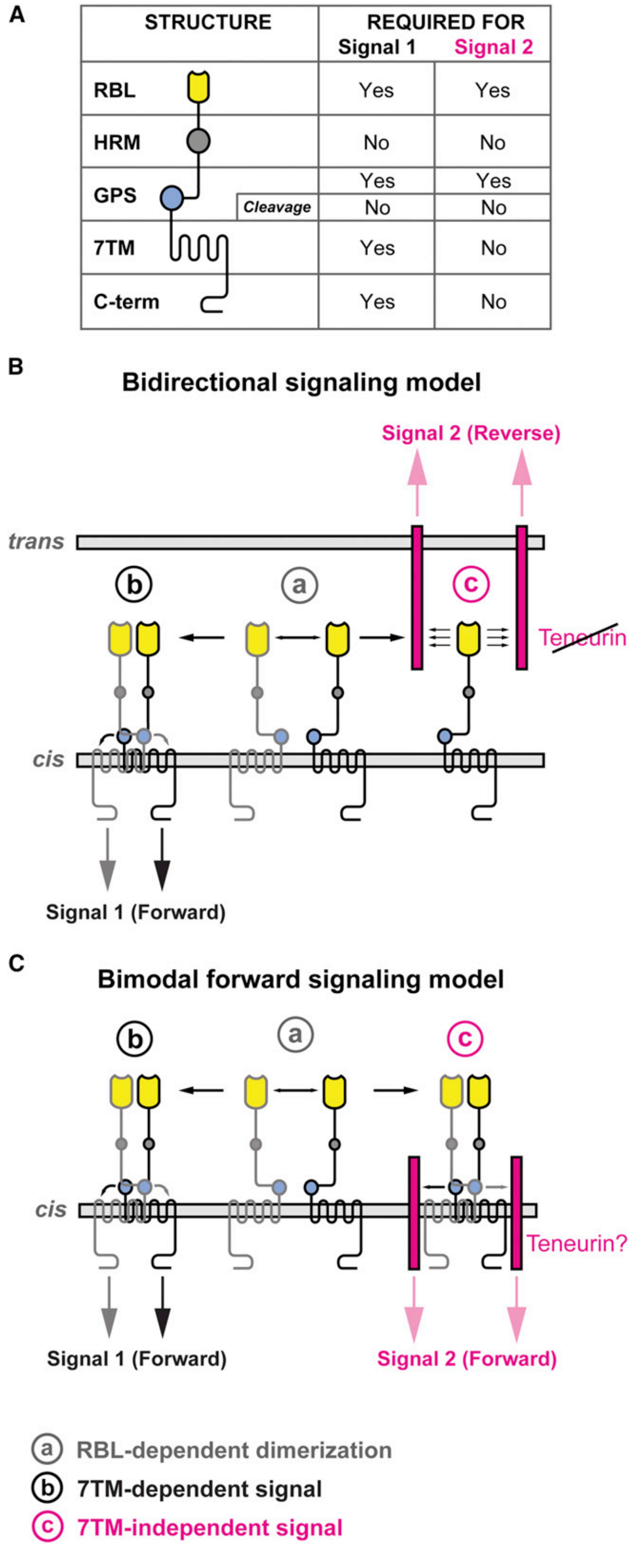

Figure 7. Models of LAT-1 Signaling

(A) Summary figure of structure-function correlation for two different activities of the LAT-1 receptor. Two models of receptor function incorporate these findings.

(B and C) Homodimerization through the RBL domain (a) initiates the 7TM-dependent LAT-1 activity (b). (B) In the bidirectional signaling model, the LAT-1 ectodomain interacts in *trans* with a molecule on the adjacent membrane through which a reverse signal (c) is conveyed to the neighboring cell. (C) The bimodal forward signaling model places LAT-1 in a *cis*-interaction with a molecule on the same cell membrane. This model accommodates a coreceptor sensing the same stimulus as the LAT-1 ectodomain and transducing a signal (c) parallel to the 7TM-dependent signal of LAT-1.

(Langenhan et al., 2009). This results in a highly reproducible zebra-like pattern on the dorsal epidermis prior to syncytial fusion of these cells. In contrast, both nonsuperficial neuroblasts and right intercalating epidermoblasts are devoid of *lat-1::gfp* expression. Using 4D microscopy we mapped *lat-1* expression to ABplaaaap daughters, ABarppaapa, and all four Caaa granddaughters (Figures 6C and 6E). Intriguingly, an independently expressed *ten-1a::gfp* promoter reporter (Mörck et al., 2010) shows expression in the same epidermal blastomeres as *lat-1::gfp* (Figures 6D and 6E) and additional lineages (ABaraax, MSapx, MSppx; data not shown) except one (Cpaaaa; Figures 6D and 6E). This implies that *lat-1* and *ten-1* act on the same cell rather than in *trans* to each other.

Taken together these results suggest that *lat-1* and *ten-1* have overlapping but not identical functions during embryonic morphogenesis and fertility, and are unlikely to act linearly as receptor and ligand in *C. elegans*.

DISCUSSION

The biological effects of aGPCR activation are involved in complex cellular traits such as developmental decisions. Thus, the limiting factor for the study of this receptor class has been the availability of informative assays. To overcome this difficulty, we have developed an in vivo assay based on the transgenic complementation of *lat-1(ok1465)*, a developmentally lethal and subfertile *lat-1* mutant strain. This assay evaluates the activity of engineered receptor variants (summarized in Figure 7A) interacting with wild-type up- and downstream components of the pathway irrespective of prior knowledge on their identity.

Our observations indicate that the HRM of LAT-1 is dispensable for receptor function in our assays. We find no evidence for a specific ligand-binding function of the LAT-1 HRM by domain deletion (ΔHRM) or domain exchange (HRM^{CDH-6}) experiments. This result is in apparent contradiction to similar receptor mutants of the *Drosophila* aGPCR FMI, where deletion of the HRM ($FMI^{\Delta HRM}$) resulted in loss of receptor activity (Kimura et al., 2006). However, this has been interpreted as the result of a nonspecific change of domain geometry in $FMI^{\Delta HRM}$ leading to a total loss of FMI function rather than a specific loss of a putative HRM ligand binding activity. In addition, molecular models based on the 3D structures of the HRM of B1/secretin-like GPCR of the GLP receptor group show that features crucial for ligand binding are not conserved in aGPCR, although hormone binding to secretin-type HRM might differ. In contrast, we find conservation of the SCR-like fold in both 7TM families suggesting that it is a structural component of the evolutionarily ancient aGPCR family, "shuffled" into the proximity of the 7TM domain

and acquired ligand binding features in B1/secretin-like GPCR (Nordström et al., 2009).

We further demonstrate that proteolysis at the GPS of LAT-1 is not necessary for receptor trafficking and activity in vivo. Previously, autoproteolytic function of the GPS, the common molecular denominator of all aGPCR class members (Bjarnadóttir et al., 2007), has been a central element in models of aGPCR maturation and signaling (Okajima et al., 2010; Lin et al., 2011). Our findings are consistent with recent pharmacological data that show intact signaling capacity of the human aGPCR GPR133 carrying similar cleavage-disrupting mutations (Bohnekamp and Schöneberg, 2011). In addition, the results are also in accord with the presence of cleaved and uncleaved forms of aGPCR on the plasma membrane of native tissue (Iguchi et al., 2008), and with data suggesting that surface expression of aGPCR can occur independently of GPS cleavage (Chang et al., 2003; Lin et al., 2004; Krasnoperov et al., 2009).

In contrast to GPS proteolysis, we observe that aGPCR signaling requires presence of a GPS as a structural element. The central finding underpinning this notion is the loss of activity in a receptor chimera carrying a heterologous GPS domain of LAT-2 in a LAT-1 context (GPS^{LAT-2}) during development. A similar effect is achieved if the 7TM domain of LAT-1 is truncated but the GPS is left intact (ΔTM2-7). This suggests that the GPS interacts with the homologous 7TM domain of LAT-1, and possibly serves as an endogenous ligand during receptor activity. We investigated this model by intermolecular complementation assays using pairs of signaling-deficient LAT-1 variants. A combination of GPS^{LAT-2} and ΔTM2-7 receptors, each deficient in rescuing developmental defects of *lat-1* mutants, reconstitutes full receptor function. This implies that homologous pairing of GPS and 7TM from two receptor molecules is required for receptor activity, which is corroborated by our biochemical evidence of LAT-1 dimers. Recent structural data of the GAIN domain, in which the GPS motif is embedded, will help to guide site-directed mutagenesis efforts to probe this putative interface in the future (Araç et al., 2012).We also provide evidence that the split personality model of aGPCR activity (Silva et al., 2009) does not account for reconstituted receptor function of hemi-receptors in the in vivo complementation assay. The biological implications of this split personality receptor model are intriguing as the putative cross-interaction of NTF and CTF derived from different aGPCR precursors could potentially re-route external signals perceived via the NTF to alternative intracellular messenger cascades activated through the CTF. The model suggests that the GPS^{LAT-2}-ΔTM2-7 reconstitution could ensue through formation of a functional receptor chimera by transfer of the NTF donated by the ΔTM2-7 to the CTF of the GPS^{LAT-2} after GPS cleavage of both parent receptors (Silva et al., 2009). Interestingly, we find that proteolysis-deficient receptor versions of either partner are sufficient to reconstitute full function in vivo. Consequently, it appears unlikely that aGPCR signaling requires domain exchange in accord with a split personality model.

The GPS^{LAT-2} chimeric receptor also uncovered separate effects of *lat-1* on fertility and tissue polarity/morphogenesis. *lat-1* is expressed in the somatic gonad (Langenhan et al., 2009), and *lat-1* mutants show reduced number of eggs laid and a high degree of sterility. Loss of fertility in *lat-1* mutants but not developmental defects are efficiently rescued by a chimeric GPS^{LAT-2} receptor. This suggests that two different activities reside in the LAT-1 receptor molecule. One requires an interaction with the 7TM domain ($LAT\text{-}1^{GPS} \leftrightarrow LAT\text{-}1^{7TM/C}$) constituting a forward signal triggered by the GPS as a tethered agonist and transduced by the 7TM. The second activity is independent of the 7TM domain ($LAT\text{-}1^{GPS} \leftrightarrow X$). Thus, the GPS interacts with partners, which are differentially affected in the chimeric GPS^{LAT-2} receptor protein. Two different models can be devised to account for the 7TM-independent function of LAT-1.

In the bidirectional signaling model a second, reverse signal is transmitted to the partner cell via engagement of the RBL domain (Figure 7B). The forward signal is disrupted in the GPS^{LAT-2} chimera, whereas the reverse signal is still present. Studies in *Drosophila* suggest that another aGPCR design, the CELSR homolog FMI/STAN, also relays bidirectional signaling states via heterologous *cis*-interactions (forward signal) (Usui et al., 1999; Chen et al., 2008; Strutt and Strutt, 2008) and homophilic *trans*-interactions (reverse signal) (Usui et al., 1999; Kimura et al., 2006; Chen et al., 2008). Our molecular analyses do not support a noncell-autonomous activity of a diffusible LAT-1 ectodomain (Langenhan et al., 2009), a separable activity of the anchored N terminus based on adhesive properties, or the activation of counter-receptors on adjacent cells in a reverse signaling mode (this work). We do not observe partial rescue through a receptor lacking the GPS (ΔGPS), where the reverse signal should still be intact. But we can currently not exclude that the overall topology of the receptor is disrupted in ΔGPS nor can we exclude that N-terminal portions of the GAIN domain, the interspaced region between HRM and GPS (Araç et al., 2012), contributes to reverse signal.

The alternative bimodal forward signaling model encompasses a second, parallel forward signal that is still active in the GPS^{LAT-2} chimera (Figure 7C). Our results are consistent with a model in which the homodimerization of the NTF could be ligand-induced through the RBL domain. This would lead to the cross-activation of the 7TM domain by the GPS of the partner molecule (Figure 7). The model is consistent with the action of the GPS as a tethered agonist of the 7TM domain (forward signal 1) and mediating protein-protein interaction with accessory proteins equivalent to RAMPs in B1/Secretin-type GPCR (forward signal 2) (Qi and Hay, 2010). An interaction candidate acting on the same cell membrane transducing the 7TM-independent activity of LAT-1, is Teneurin/*ten-1*, a putative LPHN1 ligand (Silva et al., 2011). Our experiments show coexpression of *lat-1* and *ten-1* in the developing epidermis implying that LAT-1 and TEN-1 reside in close proximity in the cell membrane, possibly as part of the same signaling complex. In addition, animals lacking *lat-1* or *ten-1* display dosage-dependent nonallelic noncomplementation. These results are not compatible with an epistatic genetic relationship expected from a ligand-receptor pair but rather indicate parallel functions for *lat-1* and *ten-1*.

Finally, future studies need to focus on the possibility that features of both signaling models are present in aGPCR. Although we have found no direct evidence for a *trans*-acting function of LAT-1 in *C. elegans*, a recent report indicates that rodent Latrophilin 1/CIRL1/CL1 interacts in *trans* with neurexins/NRX with high affinity in cell culture (Boucard et al., 2012). This interaction

requires the presence of the olfactomedin domain, which is only present in vertebrate latrophilin homologs (Figure S1B; Langenhan et al., 2009), and might exert a role in synapse formation and/or function.

In summary, we have shown that the aGPCR LAT-1 is capable of relaying at least two different signals. The GPS comprises the central structural element of aGPCR function that could be instrumental for the conversion of an adhesive event into receptor output through G protein signaling, whereas the proteolytic nature of the GPS is not required for this activity. Given the general pharmacological tractability of 7TM receptors, aGPCR are an attractive yet unexploited target to modulate adhesion-triggered cell behaviors involved in neurological and immune functions, and for tumor treatment.

EXPERIMENTAL PROCEDURES

C. elegans Strains

C. elegans strains were cultured and manipulated according to standard protocols (Brenner, 1974). Wild-type worms were *C. elegans* variety Bristol, N2. Alleles *lat-1(ok1465)* and *ten-1(ok641)* were generated by the *C. elegans* gene knockout consortium. Strains were provided by the Caenorhabditis Genetics Center, which is funded by the NIH National Center for Research Resources (NCRR). A full list of transgenic worm strains is included in the Extended Experimental Procedures.

Lethality and Fertility Rescue Assay

The adult brood size and oocyte assay was conducted as previously described (Langenhan et al., 2009). All experiments were conducted at least in triplicate. A two-tailed Mann-Whitney test of data sets against a respective wild-type control construct data set were performed using Prism 5 (GraphPad Software, La Jolla, CA).

Microscopy

For analysis of transgene expression embryos were dissected from gravid hermaphrodites and mounted in M9 onto a 2% agarose pad. Images were acquired with a Deltavision Core (Applied Precision), a Leica SP5 II confocal microscope (Leica, Milton Keynes, UK) and a Zeiss confocal LSM5 setup. Four-dimensional imaging and lineage analysis of *lat-1::gfp* and *ten-1a::gfp* expressing cells were performed as previously described using SIMI Biocell software (SIMI Reality Motion Systems, Germany) (Bischoff and Schnabel, 2006; Langenhan et al., 2009). Z-stacks with spatial spacing of 0.5 μm were taken.

Protein Biochemistry

Worms were washed off culture plates with ice-cold water, pelleted by centrifugation at 13,000 rpm for 5 min and freeze-cracked in dry ice/ethanol. The lysate was boiled in 100 μl TBS (20 mM Tris, 150 mM NaCl, pH 7.5) with 1% SDS for 10 min, spun down, and supernatant and pellet separately mixed with 2× Laemmli buffer. A detailed description of methodologies used for protein analysis including western blotting, immobilized metal ion affinity chromatography, size-exclusion chromatography by gel filtration, and analytical ultracentrifugation can be found in the Extended Experimental Procedures.

SUPPLEMENTAL INFORMATION

Supplemental Information includes Extended Experimental Procedures, four figures, and four tables and can be found with this article online at http://dx.doi.org/10.1016/j.celrep.2012.06.015.

LICENSING INFORMATION

This is an open-access article distributed under the terms of the Creative Commons Attribution 3.0 Unported License (CC-BY; http://creativecommons.org/licenses/by/3.0/legalcode).

ACKNOWLEDGMENTS

This work was supported by grants from the BBSRC (BB/C504200/1), the EPA Cephalosporin Trust (CF077), and the Royal Society, and by studentships from the Daimler-Benz Foundation (S.P.), the Wellcome Trust (T.L.), the Heinrich-Hertz-Stiftung (M.F.), and the Algerian Ministry of Higher Education and Scientific Research (L.M.); I.V. is supported by a Wellcome Trust Career Development Fellowship. T.L. acknowledges support from the Wellcome Trust (WT075336/Z/04/A), the Deutsche Forschungsgemeinschaft (La 2861/1-1), the IZKF Würzburg (Z-3/12) and Magdalen College, Oxford. The authors thank Marc Pilon for the *ten-1a::gfp* plasmid, Cathrin Struck for technical assistance, and Manfred Heckmann and Robert J. Kittel for comments on the manuscript. Author contributions: S.P., M.F., L.M., S.H., R.S., T.L. performed experiments, D.S. and I.V. contributed to biochemical and structural analyses, A.W. helped with genetic analyses, T.S. conducted bioinformatical calculations. A.P.R. conceived the project, A.P.R. and T.L. coordinated the study, S.P., A.P.R. and T.L. wrote the manuscript.

Received: March 5, 2012
Revised: May 15, 2012
Accepted: June 15, 2012
Published online: August 2, 2012

REFERENCES

Araç, D., Boucard, A.A., Bolliger, M.F., Nguyen, J., Soltis, S.M., Südhof, T.C., and Brunger, A.T. (2012). A novel evolutionarily conserved domain of cell-adhesion GPCRs mediates autoproteolysis. EMBO J. *31*, 1364–1378.

Arcos-Burgos, M., Jain, M., Acosta, M.T., Shively, S., Stanescu, H., Wallis, D., Domené, S., Vélez, J.I., Karkera, J.D., Balog, J., et al. (2010). A common variant of the latrophilin 3 gene, LPHN3, confers susceptibility to ADHD and predicts effectiveness of stimulant medication. Mol. Psychiatry *15*, 1053–1066.

Bischoff, M., and Schnabel, R. (2006). A posterior centre establishes and maintains polarity of the Caenorhabditis elegans embryo by a Wnt-dependent relay mechanism. PLoS Biol. *4*, e396.

Bjarnadóttir, T.K., Fredriksson, R., and Schiöth, H.B. (2007). The adhesion GPCRs: a unique family of G protein-coupled receptors with important roles in both central and peripheral tissues. Cell. Mol. Life Sci. *64*, 2104–2119.

Bohnekamp, J., and Schöneberg, T. (2011). Cell adhesion receptor GPR133 couples to Gs protein. J. Biol. Chem. *286*, 41912–41916.

Boucard, A.A., Ko, J., and Südhof, T.C. (2012). High affinity neurexin binding to cell adhesion G-protein-coupled receptor CIRL1/latrophilin-1 produces an intercellular adhesion complex. J. Biol. Chem. *287*, 9399–9413.

Bray, S.J. (2006). Notch signalling: a simple pathway becomes complex. Nat. Rev. Mol. Cell Biol. *7*, 678–689.

Brenner, S. (1974). The genetics of Caenorhabditis elegans. Genetics *77*, 71–94.

Chang, G.W., Stacey, M., Kwakkenbos, M.J., Hamann, J., Gordon, S., and Lin, H.H. (2003). Proteolytic cleavage of the EMR2 receptor requires both the extracellular stalk and the GPS motif. FEBS Lett. *547*, 145–150.

Chen, W.-S., Antic, D., Matis, M., Logan, C.Y., Povelones, M., Anderson, G.A., Nusse, R., and Axelrod, J.D. (2008). Asymmetric homotypic interactions of the atypical cadherin flamingo mediate intercellular polarity signaling. Cell *133*, 1093–1105.

Drabikowski, K., Trzebiatowska, A., and Chiquet-Ehrismann, R. (2005). ten-1, an essential gene for germ cell development, epidermal morphogenesis, gonad migration, and neuronal pathfinding in Caenorhabditis elegans. Dev. Biol. *282*, 27–38.

Fredriksson, R., Gloriam, D.E.I., Höglund, P.J., Lagerström, M.C., and Schiöth, H.B. (2003a). There exist at least 30 human G-protein-coupled receptors with long Ser/Thr-rich N-termini. Biochem. Biophys. Res. Commun. *301*, 725–734.

Fredriksson, R., Lagerström, M.C., Lundin, L.-G., and Schiöth, H.B. (2003b). The G-protein-coupled receptors in the human genome form five main

families. Phylogenetic analysis, paralogon groups, and fingerprints. Mol. Pharmacol. *63*, 1256–1272.

Grace, C.R.R., Perrin, M.H., DiGruccio, M.R., Miller, C.L., Rivier, J.E., Vale, W.W., and Riek, R. (2004). NMR structure and peptide hormone binding site of the first extracellular domain of a type B1 G protein-coupled receptor. Proc. Natl. Acad. Sci. USA *101*, 12836–12841.

Hamann, J., Vogel, B., van Schijndel, G.M., and van Lier, R.A. (1996). The seven-span transmembrane receptor CD97 has a cellular ligand (CD55, DAF). J. Exp. Med. *184*, 1185–1189.

Harmar, A.J. (2001). Family-B G-protein-coupled receptors. Genome Biol. *2*, reviews3013.1–reviews3013.10.

Iguchi, T., Sakata, K., Yoshizaki, K., Tago, K., Mizuno, N., and Itoh, H. (2008). Orphan G protein-coupled receptor GPR56 regulates neural progenitor cell migration via a G alpha 12/13 and Rho pathway. J. Biol. Chem. *283*, 14469–14478.

Kimura, H., Usui, T., Tsubouchi, A., and Uemura, T. (2006). Potential dual molecular interaction of the Drosophila 7-pass transmembrane cadherin Flamingo in dendritic morphogenesis. J. Cell Sci. *119*, 1118–1129.

Krasnoperov, V., Deyev, I.E., Serova, O.V., Xu, C., Lu, Y., Buryanovsky, L., Gabibov, A.G., Neubert, T.A., and Petrenko, A.G. (2009). Dissociation of the subunits of the calcium-independent receptor of alpha-latrotoxin as a result of two-step proteolysis. Biochemistry *48*, 3230–3238.

Langenhan, T., Prömel, S., Mestek, L., Esmaeili, B., Waller-Evans, H., Hennig, C., Kohara, Y., Avery, L., Vakonakis, I., Schnabel, R., and Russ, A.P. (2009). Latrophilin signaling links anterior-posterior tissue polarity and oriented cell divisions in the C. elegans embryo. Dev. Cell *17*, 494–504.

Lin, H.-H., Chang, G.-W., Davies, J.Q., Stacey, M., Harris, J., and Gordon, S. (2004). Autocatalytic cleavage of the EMR2 receptor occurs at a conserved G protein-coupled receptor proteolytic site motif. J. Biol. Chem. *279*, 31823–31832.

Lin, H.-H., Stacey, M., Yona, S., and Chang, G.-W. (2011). GPS proteolytic cleavage of adhesion-GPCRs. Adv. Exp. Med. Biol. *706*, 49–58.

Mörck, C., Vivekanand, V., Jafari, G., and Pilon, M. (2010). C. elegans ten-1 is synthetic lethal with mutations in cytoskeleton regulators, and enhances many axon guidance defective mutants. BMC Dev. Biol. *10*, 55.

Nordström, K.J.V., Lagerström, M.C., Wallér, L.M.J., Fredriksson, R., and Schiöth, H.B. (2009). The Secretin GPCRs descended from the family of Adhesion GPCRs. Mol. Biol. Evol. *26*, 71–84.

Norman, D.G., Barlow, P.N., Baron, M., Day, A.J., Sim, R.B., and Campbell, I.D. (1991). Three-dimensional structure of a complement control protein module in solution. J. Mol. Biol. *219*, 717–725.

Okajima, D., Kudo, G., and Yokota, H. (2010). Brain-specific angiogenesis inhibitor 2 (BAI2) may be activated by proteolytic processing. J. Recept. Signal Transduct. Res. *30*, 143–153.

Parthier, C., Kleinschmidt, M., Neumann, P., Rudolph, R., Manhart, S., Schlenzig, D., Fanghänel, J., Rahfeld, J.-U., Demuth, H.-U., and Stubbs, M.T. (2007). Crystal structure of the incretin-bound extracellular domain of a G protein-coupled receptor. Proc. Natl. Acad. Sci. USA *104*, 13942–13947.

Piao, X., Hill, R.S., Bodell, A., Chang, B.S., Basel-Vanagaite, L., Straussberg, R., Dobyns, W.B., Qasrawi, B., Winter, R.M., Innes, A.M., et al. (2004). G protein-coupled receptor-dependent development of human frontal cortex. Science *303*, 2033–2036.

Qi, T., and Hay, D.L. (2010). Structure-function relationships of the N-terminus of receptor activity-modifying proteins. Br. J. Pharmacol. *159*, 1059–1068.

Rivero-Müller, A., Chou, Y.-Y., Ji, I., Lajic, S., Hanyaloglu, A.C., Jonas, K., Rahman, N., Ji, T.H., and Huhtaniemi, I. (2010). Rescue of defective G protein-coupled receptor function in vivo by intermolecular cooperation. Proc. Natl. Acad. Sci. USA *107*, 2319–2324.

Runge, S., Thøgersen, H., Madsen, K., Lau, J., and Rudolph, R. (2008). Crystal structure of the ligand-bound glucagon-like peptide-1 receptor extracellular domain. J. Biol. Chem. *283*, 11340–11347.

Shima, Y., Kengaku, M., Hirano, T., Takeichi, M., and Uemura, T. (2004). Regulation of dendritic maintenance and growth by a mammalian 7-pass transmembrane cadherin. Dev. Cell *7*, 205–216.

Silva, J.-P., Lelianova, V., Hopkins, C., Volynski, K.E., and Ushkaryov, Y. (2009). Functional cross-interaction of the fragments produced by the cleavage of distinct adhesion G-protein-coupled receptors. J. Biol. Chem. *284*, 6495–6506.

Silva, J.-P., Lelianova, V.G., Ermolyuk, Y.S., Vysokov, N., Hitchen, P.G., Berninghausen, O., Rahman, M.A., Zangrandi, A., Fidalgo, S., Tonevitsky, A.G., et al. (2011). Latrophilin 1 and its endogenous ligand Lasso/teneurin-2 form a high-affinity transsynaptic receptor pair with signaling capabilities. Proc. Natl. Acad. Sci. USA *108*, 12113–12118.

Stacey, M., Chang, G.-W., Davies, J.Q., Kwakkenbos, M.J., Sanderson, R.D., Hamann, J., Gordon, S., and Lin, H.-H. (2003). The epidermal growth factor-like domains of the human EMR2 receptor mediate cell attachment through chondroitin sulfate glycosaminoglycans. Blood *102*, 2916–2924.

Strutt, H., and Strutt, D. (2008). Differential stability of flamingo protein complexes underlies the establishment of planar polarity. Curr. Biol. *18*, 1555–1564.

Usui, T., Shima, Y., Shimada, Y., Hirano, S., Burgess, R.W., Schwarz, T.L., Takeichi, M., and Uemura, T. (1999). Flamingo, a seven-pass transmembrane cadherin, regulates planar cell polarity under the control of Frizzled. Cell *98*, 585–595.

Vakonakis, I., Langenhan, T., Prömel, S., Russ, A., and Campbell, I.D. (2008). Solution structure and sugar-binding mechanism of mouse latrophilin-1 RBL: a 7TM receptor-attached lectin-like domain. Structure *16*, 944–953.

Volynski, K.E., Silva, J.-P., Lelianova, V.G., Atiqur Rahman, M., Hopkins, C., and Ushkaryov, Y.A. (2004). Latrophilin fragments behave as independent proteins that associate and signal on binding of LTX(N4C). EMBO J. *23*, 4423–4433.

Wei, W., Hackmann, K., Xu, H., Germino, G., and Qian, F. (2007). Characterization of cis-autoproteolysis of polycystin-1, the product of human polycystic kidney disease 1 gene. J. Biol. Chem. *282*, 21729–21737.

Weston, M.D., Luijendijk, M.W.J., Humphrey, K.D., Möller, C., and Kimberling, W.J. (2004). Mutations in the VLGR1 gene implicate G-protein signaling in the pathogenesis of Usher syndrome type II. Am. J. Hum. Genet. *74*, 357–366.

Yu, S., Hackmann, K., Gao, J., He, X., Piontek, K., García-González, M.A., Menezes, L.F., Xu, H., Germino, G.G., Zuo, J., and Qian, F. (2007). Essential role of cleavage of Polycystin-1 at G protein-coupled receptor proteolytic site for kidney tubular structure. Proc. Natl. Acad. Sci. USA *104*, 18688–18693.

Cell PRESS Open ACCESS

EXTENDED EXPERIMENTAL PROCEDURES

Generation of Transgenic Lines

All transgenic strains with stably transmitting extrachromosomal arrays except the *ten-1a::gfp* reporter were generated by DNA microinjection as described (Mello et al., 1991; Mello and Fire, 1995). Cosmids were injected at a concentration of 1 ng/μl together with the coinjection marker *pRF4[rol-6(su1006)+]* (100 ng/μl) and *pBluescript II SK+* vector DNA (Stratagene, La Jolla, USA) as stuffer DNA to achieve a final concentration of 120 ng/μl. For coinjections of two different cosmids, 1 ng of each was utilized and the stuffer DNA adjusted accordingly. DNA was injected into the syncytical gonad of *lat-1(ok1465)/mIn1[mIs14 dpy-10(e128)]* hermaphrodites. Transgenic progeny were isolated and stable lines selected. Multiple independent transgenic lines were established for each transgene tested. A construct was scored as not rescuing when ten independent lines did not produce transgenic homozygotes. The *ten-1a::gfp* reporter construct *pQC08.3* (Mörck et al., 2010) together with the co-transformation marker *pBx[pha-1+]* was transformed biolistically into *pha-1(e2123ts)* mutants and transgenic worms were selected based on *pha-1* rescue at the restrictive temperature (Wilm et al., 1999). For detailed description of transgene construction see below.

C. elegans Strains Used in the Study

The following extrachromosomal arrays were created for this study: *aprEx47[lat-1(1-581) (pTL20) rol-6(su1006) pBSK], aprEx52[lat-1(H528A) (pSP18) rol-6(su1006) pBSK], aprEx53[lat-1(H528A) (pSP18) rol-6(su1006) pBSK], aprEx63[lat-1(1-581; H528A) (pSP20) rol-6(su1006) pBSK], aprEx69[lat-1(T530A) (pSP19) rol-6(su1006) pBSK], aprEx73[lat-1(T530A) (pSP19) rol-6(su1006) pBSK], aprEx84 [lat-1(1-581; T530A) (pSP30) rol-6(su1006) pBSK], aprEx118[lat-1ΔGPS(483-542)::gfp (pSP43) rol-6(su1006) pBSK], aprEx130[lat-1(C497S)::gfp (pSP44) rol-6(su1006) pBSK], aprEx146[lat-1ΔHRM(179-243)::gfp (pSP68) rol-6(su1006) pBSK], aprEx147[lat-1ΔHRM(179-243)::gfp (pSP68) rol-6(su1006) pBSK], aprEx153[lat-1(H528A)::gfp (pSP53) rol-6(su1006) pBSK], aprEx157[lat-1(lat-2 GPS) (pSP75) rol-6(su1006) pBSK], aprEx158[lat-1(lat-2 GPS) (pSP75) rol-6(su1006) pBSK], aprEx159[lat-1(cdh-6 HRM)::gfp (pSP79) rol-6(su1006) pBSK], aprEx160[lat-1(cdh-6 HRM)::gfp (pSP79) rol-6(su1006) pBSK], aprEx166[lat-1(T530A)::gfp (pSP85) rol-6(su1006) pBSK], aprEx174-177 [ten-1a::gfp pha-1(+)].* The following alleles and transgenes have been previously described (Langenhan et al., 2009): *qaEx7513[lat-1(+) (pTL2) rol-6(su1006)], aprEx77[lat-1::gfp (pSP5) rol-6(su1006) pBSK], aprEx89[lat-1 (1-581) ΔGPS(483-542)::gfp (pSP36) rol-6(su1006) pBSK], qaIs7524[lat-1p::gfp (pTL13) rol-6(su1006)].*

Construction of Transgenes

For generation of constructs recombineering was employed (Dolphin and Hope, 2006; Tursun et al., 2009) and accompanying protocols were modified to construct latrophilin transgenes using cosmids, PCR-amplified targeting cassettes, and positive antibiotic selection.

Constructs pTL2 and pSP5 (Langenhan et al., 2009) were used as a basis to construct modified versions with the recombination-competent *E. coli* strain SW105 developed by Copeland and co-workers (Warming et al., 2005) (http://recombineering.ncifcrf.gov/) containing the genes of the λ-Red recombinase system under the control of a temperature-sensitive repressor that restricts recombination activity under normal growth conditions at 32°C. To induce recombinase expression, culture temperature was increased to 42°C for 15 min before making the bacteria electrocompetent for transformation.

The cosmid to be modified was transformed into one of the strains, the recombinase system was induced and the bacteria were made electrocompetent again. Modification of the cosmid was conducted via a targeting cassette containing the desired modification and a kanamycin-resistance gene (*kanR*) under a prokaryotic promoter as a selection marker. These two were flanked by at least 40 bp of homology to the region where the cosmid should be modified. This targeting cassette was generated using different approaches depending on the modification, and then transformed into the electrocompetent bacteria containing the cosmid in which the recombination occurred. Cells with recombined cosmids were kanamycin-resistant and were selected on LB agar with kanamycin and grown in LB culture shaking at 32°C overnight. Cosmids were verified by restriction digest and sequencing.

lat-1(179-243)::gfp (pSP68) = ΔHRM

To generate the deletion targeting cassette a *loxP-kanR-loxP* cassette was amplified from vector PL452 (Liu et al., 2003) with primers lat1_282F/lat1_283R (for primer sequences see Table S4) introducing at the 3′ end via the primer a sequence homologous to the last 10 bases of intron 4 and to exon 5 until the HRM of *lat-1*. A 341 bp fragment of the region downstream of the HRM was amplified using primers lat1_284F/lat1_285R and ligated to the also gel-purified *loxP-kanR-loxP* cassette. To ensure ligation of this fragment 5′ of the cassette, the forward primer of this fragment and the reverse primer of the cassette were phosphorylated beforehand. Ligation was conducted using an 1: 1 molar ratio of both fragments. 2 μl of the ligation product were used in a PCR amplifying the complete ligated fragment applying primers rec_138F/lat1_285R with overhangs homologous to pSP5. The resulting cassette was recombineered into pSP5 and the selection cassette subsequently removed.

lat-1(cdh-6 HRM)::gfp (pSP79) = HRM^{CDH-6}

Construct pSP79 is based on pSP5. The targeting cassette consisted of two parts. *A loxP-kanR-loxP* cassette was amplified from vector PL452 using primers lat1_282F/lat1_283R introducing at the 3′ end via the forward primer a sequence homologous to the last 10 bases of intron 4 and to exon 5 until the HRM of *lat-1*. The HRM of *cdh-6* (aa 1913 - 1975) was amplified using primers

lat1_327F/lat1_328R from a *C. elegans* cDNA library. The reverse primer of the *loxP-kanR-loxP* cassette and the forward primer of the *cdh-6* HRM were phosphorylated beforehand. Both fragments were ligated using an 1: 1 molar ratio. 2 μl of the ligation product were used in a PCR amplifying the complete ligated fragment applying primers rec_138F/rec_141R containing overhangs homologous to pSP5, the PCR product gel-purified and recombineered into pSP5. The selection cassette subsequently removed.

lat-1(1-581; pat-3 TM) (pSP38) = TMΔ2-7/TM^{PAT-3}

Replacement of the first transmembrane domain of LAT-1 with the one of the β-integrin PAT-3 (GenBank acc. no. Z35604.2 GI:19571719) was performed using a two-step strategy. A sequence of *pat-3* corresponding to aa 738 – 760 (bp 2214 – 2280) was synthesized by Geneart (Berlin, Germany) together with the sequence upstream the first transmembrane domain corresponding to aa 509 – 549 (bp 1527 – 1647) 5′ of the *pat-3* sequence. Synthesis product and the vector pPolycistronic were cut with *Age*I and *Nco*I, gel-purified, ligated and transformed into chemically competent *E. coli* TOP10 cells. The subcloned fragment, the *gfp* and the *kanR* cassette from pPolycistronic were amplified with primers lat1_216F/lat1_217R with the reverse primer containing a homologous sequence to the 3′ UTR of *lat-1*, gel-purified and recombineered into pTL2.

lat-1(37-542, T530A) (pSP32) = LAT-1(37-542)$^{GPS(T530A)}$::6xHis

The *lat-1* GPS and N-terminus sequences were amplified from *C. elegans* cDNA using primers lat1_190F/lat1_191R. Forward primers contained an *Age*I site and reverse primers a KpnI site and the GPS T530A mutation changing codon ACA to GCA. Fragments were gel-purified, digested with *Age*I and KpnI, purified using a QIAquick PCR purification kit and ligated into the vector pHLSeq, which was also cut with *Age*I and KpnI and gel-purified. The ligation mix was transformed into chemically competent *E. coli* TOP10 cells (Invitrogen, Paisley, UK) according to manufacturer's instructions.

lat-1(C497S)::gfp (pSP44) = GPS$^{C > S}$

To generate the point mutation targeting cassette for pSP44, a 450 bp fragment of exon 5 was amplified from pTL2 using primers lat1_03F/lat1_218R introducing to codon change TGT > AGT with the reverse primer. The selection-cassette was amplified together with 0.7 kb of exon 5 upstream of the cassette from pSP34 using primers lat1_219F lat1_220R (intermediate cosmid with *FRT-kanR-FRT* [not shown]). Reverse primer of the first and forward of the latter were phosphorylated beforehand. Both fragments were ligated using an 1:1 molar ratio. 3 μl of the ligation product were used in a PCR amplifying the complete ligated fragment applying primers lat1_03F/lat1_220R with overhangs homologous to pSP5, the PCR product was transformed in electrocompetent, recombinase-induced SW105 cells containing pSP5 for recombineering. The resulting cassette was recombineered into pSP5 and the selection cassette subsequently removed.

lat-1(H528A) (pSP18) = GPS$^{H > A}$, lat-1(T530A) (pSP19) = GPS$^{T > A}$, lat-1(H528A)::gfp::lat-1 (pSP53) = GPS$^{H > A}$, lat-1(T530A)::gfp::lat-1 (pSP85) = GPS$^{T > A}$

Cosmids pSP53 and pSP85 are based on pSP5, cosmids pSP18 and pSP19 are based on pTL2. The targeting cassette consisted of two parts. A 0.2 kb fragment of exon 5 was amplified from pTL2 with forward primers introducing a point mutation. For H528A the codon CAT was changed to GCT (primers lat1_150F/lat1_149R), for T530A the codon ACA was changed to GCA (primers lat1_148F/ lat1_149R). The reverse primer contained an overhanging *Hind*III site. The second part of the cassette was the *FRT-kanR-FRT* cassette amplified from pIGCN21 (Lee et al., 2001) (primers lat1_137F/lat1_138R) with the forward primer containing an overhanging HindIII site. Both fragments were, cut with *Hind*III and ligated using an 1: 1 molar ratio. 2 μl of the ligation product were used in a PCR amplifying the complete fragment applying primers rec_97F/rec_96R or rec_95F/rec_96R with overhangs homologous to pSP5/ pTL2. The resulting cassettes were recombineered into pSP5/pTL2 and the selection cassettes subsequently removed.

lat-1(1-581;H528A)::gfp (pSP20) = GPS$^{H > A}$/ΔTM2-7, lat-1(1-581;T530A)::gfp (pSP30) = GPS$^{T > A}$/ΔTM2-7

Cosmids pSP20 and pSP30 are based on pTL20 and generated by using the same strategy (Langenhan et al., 2009). A 4.2 kb BamHI-fragment was released from pTL20 containing exon 4, exon 5 and the *gfp::kan* cassette as well as the 3′UTR of *lat-1*. This fragment was gel-purified and used as a targeting cassette for recombineering into the corresponding full length construct: into pSP18 to generate pSP20 and into pSP19 to generate pSP30.

lat-1(483-542)::gfp (pSP43) = ΔGPS

The targeting cassette deleting the sequence corresponding to aa 483 – 542 of LAT-1 was generated by fusion of two fragments. First, a 421 bp fragment containing the sequence 5′ of the GPS was amplified from pTL2 (primers lat1_212F/lat1_213R [phosphorylated]). Second, the sequence 3′ of the GPS followed by the selection-cassette in intron 5 was amplified from pSP34 (intermediate with *FRT-kanR-FRT*, not shown) with primers lat1_214F (phosphorylated)/lat1_221R. Both fragments were ligated using an 1:1 molar ratio. 3 μl of the ligation product were used in a PCR amplifying the complete ligated fragment applying primers with overhangs homologous to pSP5 (lat1_212F/lat1_221R). The resulting cassette was recombineered into pSP5 and the selection cassette subsequently removed.

lat-1(lat-2 GPS)::gfp (pSP75) = GPS^{LAT-2}

In cosmid pSP75, the LAT-1 GPS (aa 483-542) is exchanged for the LAT-2 GPS (aa 837-886). The *lat-2* GPS was amplified from a *C. elegans* cDNA library using primers lat1_247F/lat1_248R, the selection cassette was amplified from pSP34 (intermediate with *FRT-kanR-FRT* (not shown)) using primers lat1_244F/lat1_246R for recombineering into pSP5. The reverse primer of the first and forward primer of the second fragment were phosphorylated. Both fragments were ligated using an 1:1 molar ratio. 3 μl of the ligation product were used in a PCR amplifying over the complete length of the ligation product using primers rec_135F/rec_136R for recombineering into pSP5. The selection cassette subsequently removed.

lat-1(1-795)::gfp (pSP21) = ΔCterm

To yield construct pSP21, the *gfp::kanR* cassette of cosmid pTL20 was amplified using primers rec_102F/rec_103 inserting five alanines with the forward primer. Fragments were gel-purified and transformed into electrocompetent SW105 cells for recombineering.

lat-1(lat-2 GPS^{T530A})::gfp::lat-1 (pSP97) = LAT-2$^{GPS(T>A)}$

This cosmid is based on pSP75. The targeting cassette consisted of two parts. A 0.2 kb fragment around the GPS was amplified from pSP75 (primers lat1_335F/lat1_336R) with the forward primer introducing a point mutation. The codon ACA was changed to GCA. The reverse primer contained an overhanging *Hind*III site. The second part of the cassette was the *loxP-kanR-loxP* amplified from vector PL452 (primers lat1_337F/lat1_338R) with the forward primer containing an overhanging *Hind*III site. Both fragments were cut with *Hind*III and ligated. The ligation product were used in a PCR amplifying the complete fragment applying primers with overhangs homologous to pSP75 (lat1_335F/lat1_338R). The PCR product was gel-purified and recombineered into pSP75. The selection cassette subsequently removed.

Normalization of Data Sets for Rescue and Epistatic Interaction

Lethality rescue

$$\overline{xn} = \frac{1}{n}\sum_{i=1}^{n} xn_i$$

where

$$xn = \frac{xr - m_{lat-1(ok1465)}}{m_{lat-1(+)}}$$

where

xn is the normalized value,
xr is the raw value,
$m_{lat-1(ok1465)}$ is the mean of the *lat-1(-)* mutant population,
$m_{lat-1(+)}$ is the mean of the population injected with the corresponding wild-type construct.

Fertility rescue

$$\overline{xn} = \frac{1}{n}\sum_{i=1}^{n} xn_i$$

where

$$xn = \frac{xr}{m_{lat-1(+)}}$$

where

xn is the normalized value,
xr is the raw value,
$m_{lat-1(+)}$ is the mean of the population injected with the corresponding wild-type construct.

FM4-64 Costaining

Plasma membrane co-staining of transgenic worm strains carrying LAT-1::GFP variants was performed using the styryl dye FM4-64 (Mohler et al., 1998; Hardin et al., 2008). Adult transgenic worms were incubated in 10 mM NaN_3 for 15 min and cut after the

pharyngeal-intestinal valve with two 27 gauge syringe needles. Anterior worm halves containing the pharynx and the nerve ring were then immersed for 5 min in dying solution containing 10 μg/ml FM4-64 (Cat. no. T3166; Molecular Probes, Eugene, USA) in Dent's saline, washed once for 5 min in Dent's saline and mounted on 1% agar pads. Two channel confocal image stacks with a *z* spacing of 1 μm of the pharynx and nerve ring were collected with an inverted Zeiss LSM510 confocal microscope with a Plan-Apochromat x63/1.4 oil immersion DIC objective and a 2x digital zoom, using the 488 nm argon and 543 nm helium-neon laser lines for excitation of GFP and FM4-64, respectively. Similar laser intensities (488 nm: 36%; 543 nm: 45%), pinhole size (optical slice thickness: 1 μm) and gain settings were used for all specimens. All images were saved as 512x512 pixel-images at 8 bit resolution.

Signal intensity in an image region outside the specimen was determined for each channel of the stacks and subtracted from the entire image intensity using ImageJ to correct for background signal. FM4-64 data were false-colored in magenta the render the images accessible for red-green color-blind readers.

Quantitative Colocalization of GFP/FM4-64 Signals via Intensity Correlation Analysis

Quantification of spatial overlay of signals in both channels of LAT-1::GFP/FM4-64 stacks were analyzed with the intensity correlation method developed by Stanley and colleagues as described in (Li et al., 2004) using the WCIF ImageJ bundle software v1.37a (University Health Network Research, Toronto, Canada; http://www.uhnresearch.ca/facilities/wcif/fdownload.html). In brief, ROI in a selected plane from non-saturated GFP/FM4-64 confocal image stacks corrected for the mean background staining (see above) of each rescuing LAT-1::GFP variant were analyzed using the 'Intensity correlation analysis' ImageJ plugin. To render the spatial relationship of GFP and FM4-64 signals in individuals within and between different transgenic worms strains comparable, FM4-64 staining and embedding was performed for all strains under identical conditions, and similar cellular profiles in the terminal bulb were selected for colocalization analyses. Intensity correlation quotients (ICQ) were calculated for each individual ROI. ICQ values reflect whether staining intensities are associated in segregated ($-0.5 < ICQ \leq 0$), random ($ICQ = \sim 0$) or dependent ($0.5 \geq ICQ > 0$) manner. Statistical analysis of ICQ values via a two-tailed Mann-Whitney test was performed with Prism 5.0a (GraphPad Software Inc., San Diego, USA).

Western Blot Analysis

Whole worm lysate was subject to electrophoresis in a 12.5% SDS-PAGE gel and transferred to a polyvinylidene difluoride membranes (Hybond P, Amersham). Blots were then probed with the primary antibody mouse anti-GFP (JL-8, Clontech) 1:2,000 overnight at 4°C. After washing, membranes were incubated for 1 hr at room temperature with horseradish-peroxidase-conjugated horse anti-mouse (Vector) 1:10,000. Western blots were developed by an enhanced chemiluminescence (ECL) detection system (Amersham). For detection of actin as loading control, membranes were stripped in Stripping buffer (1% SDS, 0.1 M Tris pH 6.8, 0.175% β-mercaptoethanol) for 30 min at 50°C, blocked and probed with mouse anti-actin (Chemicon) 1:10,000 and then incubated with horseradish-peroxidase-conjugated horse anti-mouse (Vector) 1:10,000 as described above.

Immobilized Metal Ion Affinity Chromatography

Proteins expressed in HEK293 cells tagged with six histidines at the C terminus were purified using a Co^{2+} affinity matrix. Secreted protein was harvested 72 hr post transfection. Conditioned medium was spun down, filtered through a 0.45 μm filter (Millipore, Durham, UK) and applied onto an Econo column (Biorad, Hemel Hempstead, UK) with Talon Metal affinity resin (TaKaRa, Saint-Germain-en-Laye, France). The flow through was aided by gravity. The column was washed with 150 mM NaCl, 20 mM Na_2HPO_4 pH 7.4. Protein was eluted with 500 mM imidazole in Dulbecco's Phosphate Buffered Saline without Ca^{2+} and Mg^{2+}.

Size-Exclusion Chromatography by Gel Filtration

For separation and purification of proteins the gel filtration column Superdex 75 HiLoad26/60 (Amersham, Little Chalfont, UK) with a column volume of 320 ml connected to fast protein liquid chromatography (FPLC) system (ÄKTA, Amersham, Little Chalfont, UK) was used. Prior to gel filtration, the column was equilibrated overnight with PBS (Sigma Aldrich, Poole, UK). Proteins were loaded in PBS which was also used for rinsing the column. The flow rate was 1 ml/min, elution of proteins was monitored by spectroscopy at a wavelength of 254 nm.

Analytical Ultracentrifugation

Analytical ultracentrifugation was performed with a Beckman Coulter Optima XL-I analytical ultracentrifuge equipped with scanning absorbance optics. 20 μM – 60 μM protein was studied under native conditions in PBS. UV absorbance was monitored at 280 nm. The duration of the run was 48 hr at room temperature at 17,000 rpm and 20,000 rpm, respectively. The data were fitted to an ideal monodisperse model using the program Origin (OriginLab) with a partial specific volume of 0.73 $cm^3\ g^{-1}$ and a solvent density of 1.0054 $g\ cm^{-3}$.

Ka/Ks Ratio Analysis of GPS

The rate of non-synonymous substitutions (Ka) to the rate of synonymous substitutions (Ks) was determined from nucleotide sequences (138 base pairs) of the GPS of *Lphn2*, *Celsr1* and *Celsr2* from 17 species (see Figure S4). Analyses were performed with and without the coding sequence for the cleavage site (HXS/T). Data are presented as means of Ka/Ks ratios from pairwise

comparisons using DnaSp v5 (Librado and Rozas, 2009). For statistical analysis paired t tests were performed on the basis of Ka/Ks ratios from matching species pairs.

Molecular Modeling of the LAT-1 HRM

3D structures of the HRM of human GLP1R were based on previously published data set (PDB: 3c59) (Runge et al., 2008). The structural model of the *C. elegans* LAT-1 HRM was based on 3c59 coordinates and generated by Phyre2 (Kelley and Sternberg, 2009).

SUPPLEMENTAL REFERENCES

Bjarnadóttir, T.K., Fredriksson, R., Höglund, P.J., Gloriam, D.E., Lagerström, M.C., and Schiöth, H.B. (2004). The human and mouse repertoire of the adhesion family of G-protein-coupled receptors. Genomics *84*, 23–33.

Dolphin, C.T., and Hope, I.A. (2006). *Caenorhabditis elegans* reporter fusion genes generated by seamless modification of large genomic DNA clones. Nucleic Acids Res. *34*, e72.

Hardin, J., King, R., Thomas-Virnig, C., and Raich, W.B. (2008). Zygotic loss of ZEN-4/MKLP1 results in disruption of epidermal morphogenesis in the C. elegans embryo. Dev. Dyn. *237*, 830–836.

Kelley, L.A., and Sternberg, M.J.E. (2009). Protein structure prediction on the Web: a case study using the Phyre server. Nat. Protoc. *4*, 363–371.

Langenhan, T., Prömel, S., Mestek, L., Esmaeili, B., Waller-Evans, H., Hennig, C., Kohara, Y., Avery, L., Vakonakis, I., Schnabel, R., and Russ, A.P. (2009). Latrophilin signaling links anterior-posterior tissue polarity and oriented cell divisions in the C. elegans embryo. Dev. Cell *17*, 494–504.

Lee, E.C., Yu, D., Martinez de Velasco, J., Tessarollo, L., Swing, D.A., Court, D.L., Jenkins, N.A., and Copeland, N.G. (2001). A highly efficient Escherichia coli-based chromosome engineering system adapted for recombinogenic targeting and subcloning of BAC DNA. Genomics *73*, 56–65.

Li, Q., Lau, A., Morris, T.J., Guo, L., Fordyce, C.B., and Stanley, E.F. (2004). A syntaxin 1, Galpha(o), and N-type calcium channel complex at a presynaptic nerve terminal: analysis by quantitative immunocolocalization. J. Neurosci. *24*, 4070–4081.

Librado, P., and Rozas, J. (2009). DnaSP v5: a software for comprehensive analysis of DNA polymorphism data. Bioinformatics *25*, 1451–1452.

Liu, P., Jenkins, N.A., and Copeland, N.G. (2003). A highly efficient recombineering-based method for generating conditional knockout mutations. Genome Res. *13*, 476–484.

Mello, C., and Fire, A. (1995). DNA transformation. Methods Cell Biol. *48*, 451–482.

Mello, C.C., Kramer, J.M., Stinchcomb, D., and Ambros, V. (1991). Efficient gene transfer in C.elegans: extrachromosomal maintenance and integration of transforming sequences. EMBO J. *10*, 3959–3970.

Mohler, W.A., Simske, J.S., Williams-Masson, E.M., Hardin, J.D., and White, J.G. (1998). Dynamics and ultrastructure of developmental cell fusions in the Caenorhabditis elegans hypodermis. Curr. Biol. *8*, 1087–1090.

Mörck, C., Vivekanand, V., Jafari, G., and Pilon, M. (2010). C. elegans ten-1 is synthetic lethal with mutations in cytoskeleton regulators, and enhances many axon guidance defective mutants. BMC Dev. Biol. *10*, 55.

Nordström, K.J.V., Lagerström, M.C., Wallér, L.M.J., Fredriksson, R., and Schiöth, H.B. (2009). The Secretin GPCRs descended from the family of Adhesion GPCRs. Mol. Biol. Evol. *26*, 71–84.

Runge, S., Thøgersen, H., Madsen, K., Lau, J., and Rudolph, R. (2008). Crystal structure of the ligand-bound glucagon-like peptide-1 receptor extracellular domain. J. Biol. Chem. *283*, 11340–11347.

Schiöth, H.B., and Fredriksson, R. (2005). The GRAFS classification system of G-protein coupled receptors in comparative perspective. Gen. Comp. Endocrinol. *142*, 94–101.

Tursun, B., Cochella, L., Carrera, I., and Hobert, O. (2009). A toolkit and robust pipeline for the generation of fosmid-based reporter genes in C. elegans. PLoS ONE *4*, e4625.

Warming, S., Costantino, N., Court, D.L., Jenkins, N.A., and Copeland, N.G. (2005). Simple and highly efficient BAC recombineering using galK selection. Nucleic Acids Res. *33*, e36.

Wilm, T., Demel, P., Koop, H.U., Schnabel, H., and Schnabel, R. (1999). Ballistic transformation of *Caenorhabditis elegans.* Gene *229*, 31–35.

Cell PRESS
Open ACCESS

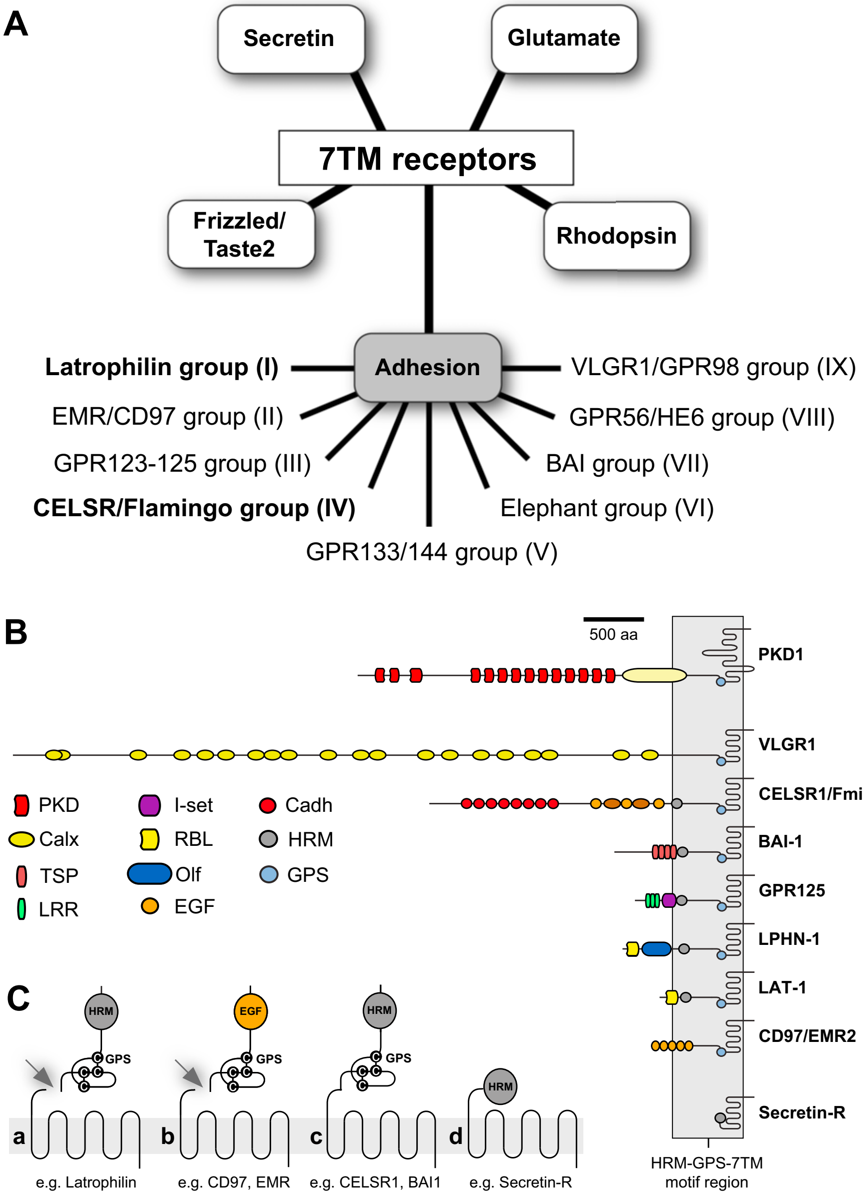

Figure S1. Organization of the 7TM Receptor Superfamily and Protein Domain Composition in Selected aGPCR Members, Related to Figure 1.

(A) 7TM receptors can be subgrouped according to the GRAFS classification (Schiöth and Fredriksson, 2005). aGPCR constitute the second largest 7TM receptor class with more than 30 members in mammalian species, which can be further classified in eight groups (Bjarnadóttir et al., 2004). Only latrophilin and CELSR/Flamingo aGPCR (bold) are conserved in invertebrate genomes (Nordström et al., 2009).

(B) Representative members of the main aGPCR groups with positions of protein domains in the N-terminus drawn to scale. Note the distance similarity of the HRM-GPS-7TM motif region (boxed in gray) among different aGPCRs. B1/Secretin receptors (bottom) contain only a HRM but no GPS, whereas the PKD1 protein (top) possesses a similar juxtamembrane GPS-7TM design as aGPCRs.

(C) aGPCR possess a GPS (a-c) in different domain contexts: cleavable GPS-HRM (a); cleavable GPS-(no HRM)-EGF (b); non-cleavable GPS-HRM (c). B1/secretin-like receptor for comparison (d).

Cell PRESS
Open ACCESS

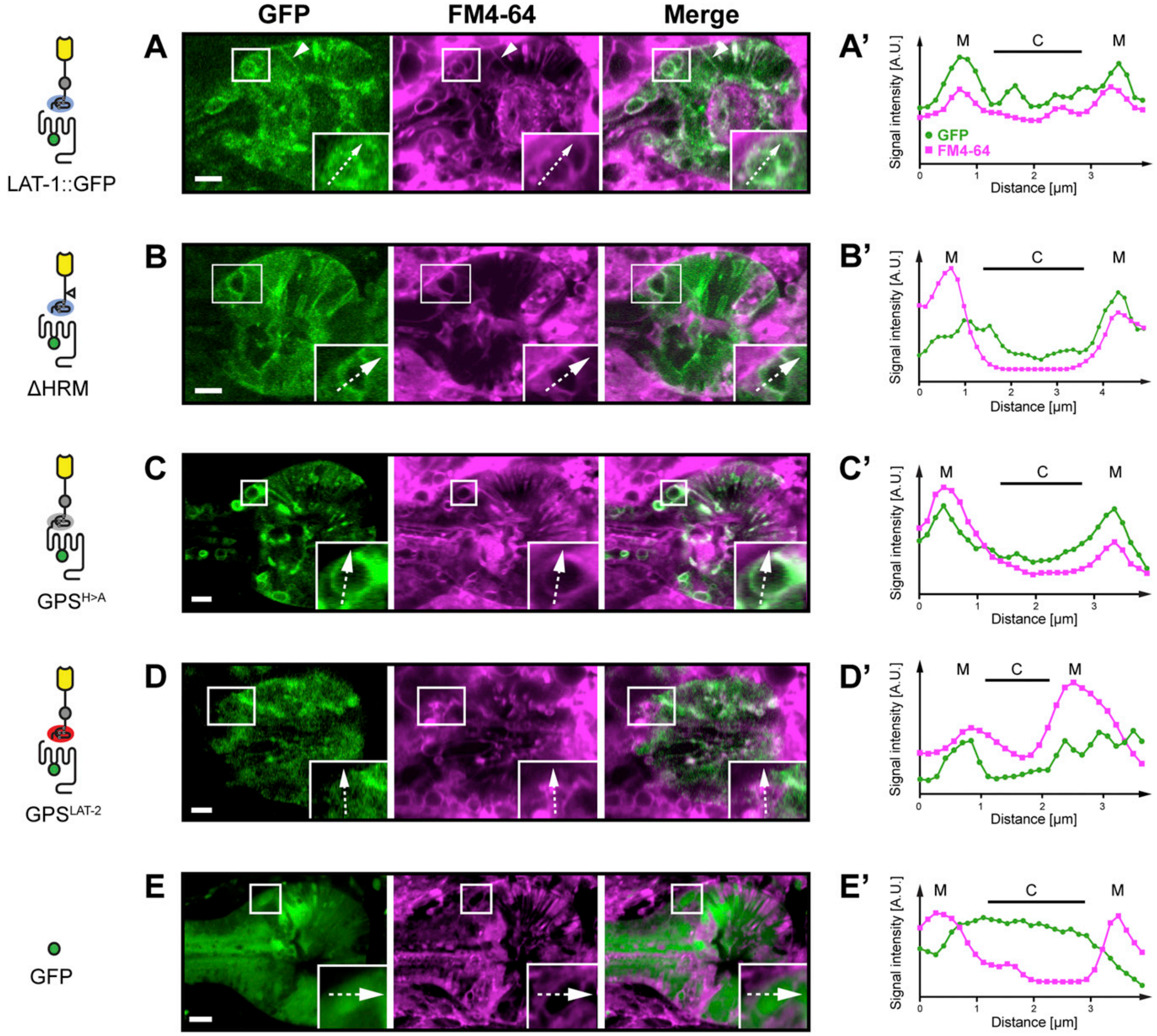

Figure S2. Membrane Targeting of LAT-1::GFP Receptor Variants Is Not Impaired, Related to Figures 2 and 4.

(A–D) Confocal images show comparable sections of the terminal bulb of the adult pharynx; anterior to the left. LAT-1::GFP variants colocalize with the membrane marker FM4-64. Inset shows higher magnification of the boxed cell. Dotted arrow indicates direction and length of plot axis in A'-D'.

(A'–D') Sectional line plots of signal intensity profiles of both channels underscores co-localization of LAT-1::GFP fusion proteins and FM4-64 intensity peaks at the membrane (M) and low signal intensities in the cytoplasm (C).

(E) A signal generated by a soluble GFP does not overlap with the membrane marker FM4-64. Inset: higher magnification of the boxed cell. Dotted arrow indicates direction and length of plot axis in E''.

(E') Sectional line plot of the signal intensity profile of both channels shows segregated intensity peaks for FM4-64 at the plasma membrane (M) and of *lat-1p*::GFP in the cytosol (C) indicating that both labels do not colocalize. Scale bars = 5 μm.

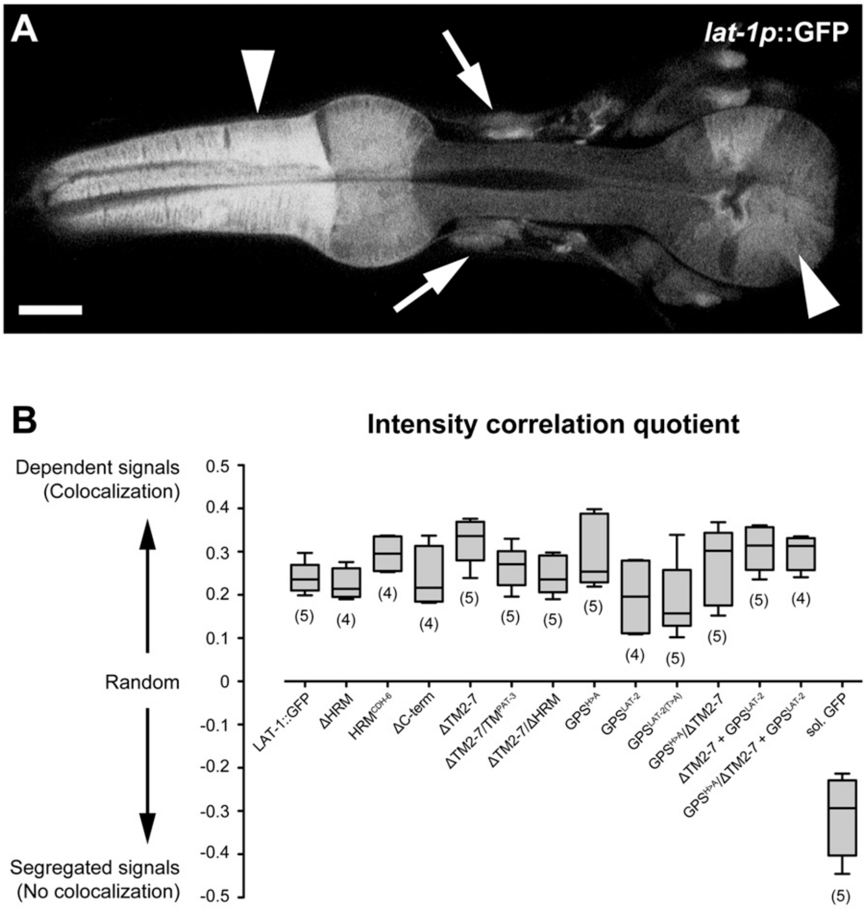

Figure S3. Quantitative Analysis of Membrane Targeting of LAT-1::GFP Receptor Variants, Related to Figures 2 and 4.

(A) Confocal section through the head region of an adult worm expressing a soluble GFP chromophore under the identical *lat-1* promoter and regulatory elements which control expression of all assayed LAT-1::GFP fusion proteins. Strong GFP expression can be seen in pharyngeal muscle (arrowheads) and the nervous system (arrows). Scale bar = 10 μm.

(B) Intensity correlation analysis of LAT-1::GFP receptor variants/FM4-64 costains. All assayed fusion proteins show dependent (i.e., colocalized) staining with the membrane marker, which is statistically indistinguishable from the LAT-1::GFP control fusion. This indicates that molecular manipulations did not disrupt membrane targeting, whereas the soluble GFP control shows segregation (i.e., non-colocalization) from the FM4-64 signal. Numbers of analyzed individuals per transgenic strain in brackets. Bold horizontal lines in the box plot represent the medians, boxes the 25% and 75% quartiles, and whiskers the minimum and maximum values of the dataset.

Cell PRESS Open ACCESS

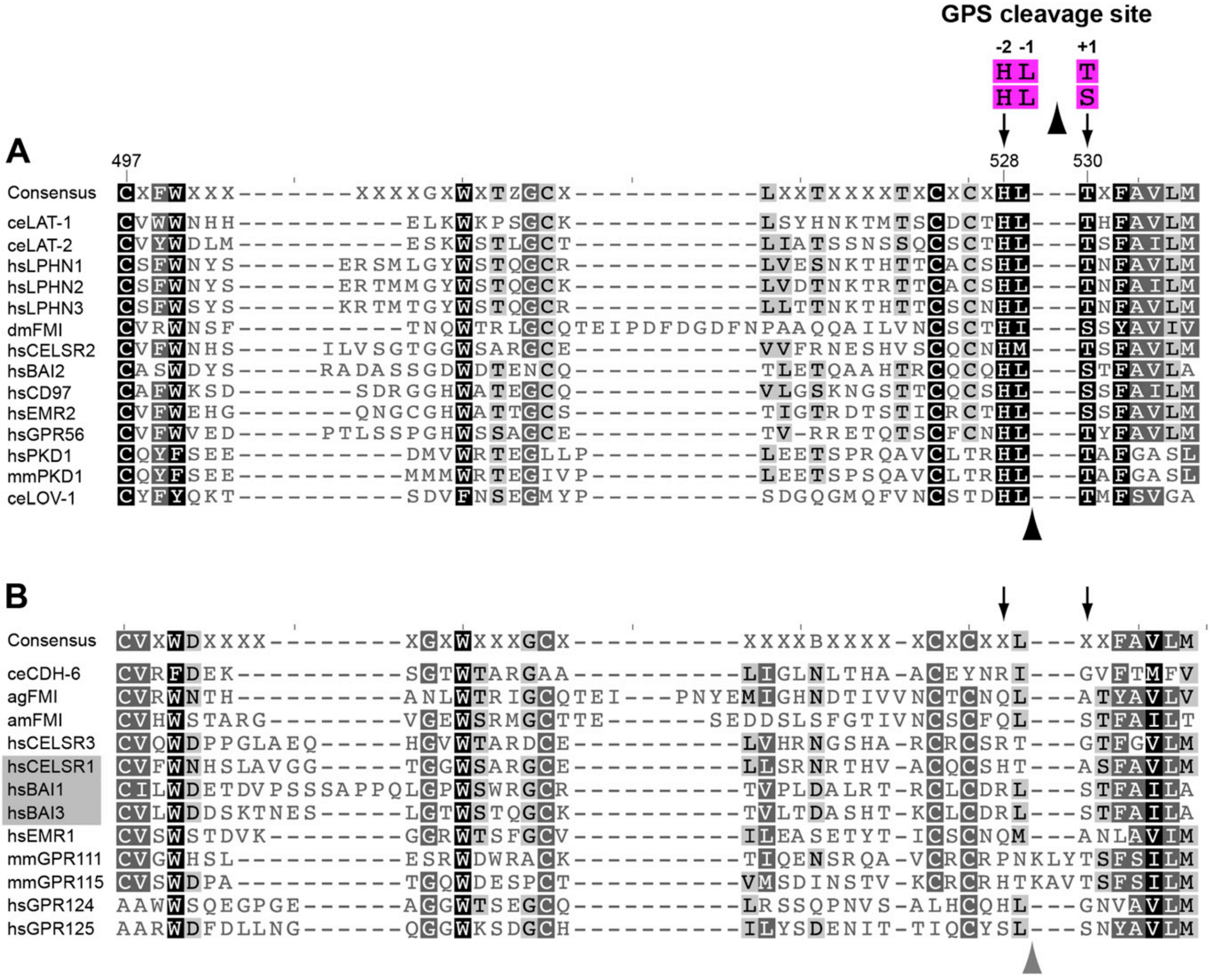

Figure S4. Presence but Not Cleavage of the GPS Is Conserved in Most aGPCRs, Related to Figure 4.

(A) Alignment of GPS with complete consensus sequence for autocatalytic cleavage. The His residue at position −2 and the S/T residues at position +1 relative to cleavage (arrowhead) are marked with arrows. The Cys residue mutated in GPR56 is in position 497 of the alignment. Residue numbering is according to the LAT-1A protein sequence.

(B) Alignment of GPS that do not conform to the consensus sequence for autocatalytic cleavage. Note that several members of the *CELSR/Fmi* family in insects, which are orthologous to the cleavable *Drosophila* FMI, do not possess the consensus cleavage motif. Loss of cleavage activity has been experimentally confirmed for GPS of aGPCR shaded in gray. Species abbreviations: ag/Anopheles gambiae, am/Apis mellifera, ce/*Caenorhabditis elegans*, dm/*Drosophila melanogaster*, hs/*Homo sapiens*, mm/*Mus musculus*.
